# Circular RNA circARPC1B functions as a stabilisation enhancer of Vimentin to prevent high cholesterol‐induced articular cartilage degeneration

**DOI:** 10.1002/ctm2.1415

**Published:** 2023-09-22

**Authors:** Jiarui Li, Xiang Li, Shengji Zhou, Yuxin Wang, Tiantian Ying, Quan Wang, Yizheng Wu, Fengchao Zhao

**Affiliations:** ^1^ Department of Orthopaedic Surgery, The First Affiliated Hospital Zhejiang University School of Medicine Hangzhou China; ^2^ Department of Orthopaedic Surgery, Sir Run Run Shaw Hospital, Zhejiang University School of Medicine Hangzhou China

**Keywords:** cholesterol, circARPC1B, osteoarthritis, Vimentin

## Abstract

**Background:**

Osteoarthritis (OA) is a prevalent and debilitating condition, that is, directly associated with cholesterol metabolism. Nevertheless, the molecular mechanisms of OA remain largely unknown, and the role of cholesterol in this process has not been thoroughly investigated. This study aimed to investigate the role of a novel circular RNA, circARPC1B in the relationship between cholesterol and OA progression.

**Methods:**

We measured total cholesterol (TC) levels in the synovial fluid of patients with or without OA to determine the diagnostic role of cholesterol in OA. The effects of cholesterol were explored in human and mouse chondrocytes in vitro. An in vivo OA model was also established in mice fed a high‐cholesterol diet (HCD) to explore the role of cholesterol in OA. RNAseq analysis was used to study the influence of cholesterol on circRNAs in chondrocytes. The role of circARPC1B in the OA development was verified through circARPC1B overexpression and knockdown. Additionally, RNA pulldown assays and RNA binding protein immunoprecipitation were used to determine the interaction between circARPC1B and Vimentin. CircARPC1B adeno‐associated virus (AAV) was used to determine the role of circARPC1B in cholesterol‐induced OA.

**Results:**

TC levels in synovial fluid of OA patients were found to be elevated and exhibited high sensitivity and specificity as predictors of OA diagnosis. Moreover, elevated cholesterol accelerated OA progression. CircARPC1B was downregulated in chondrocytes treated with cholesterol and played a crucial role in preserving the extracellular matrix (ECM). Mechanistically, circARPC1B is competitively bound to the E3 ligase synoviolin 1 (SYVN1) binding site on Vimentin, inhibiting the proteasomal degradation of Vimentin. Furthermore, circARPC1B AAV infection alleviates Vimentin degradation and OA progression caused by high cholesterol.

**Conclusions:**

These findings indicate that the cholesterol‐circARPC1B‐Vimentin axis plays a crucial role in OA progression, and circARPC1B gene therapy has the opportunity to provide a potential therapeutic approach for OA.

## Graphical abstract

High cholesterol inhibits vimentin protein expression and aggravates OA progression. High cholesterol inhibits circARPC1B expression. CircARPC1B functions as a stabilisation enhancer of vimentin to suppress chondrocytes degradation. The cholesterol/circARPC1B/Vimentin axis plays an important role in OA progression.

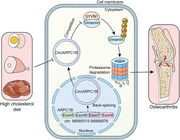

## 1 INTRODUCTION

Osteoarthritis (OA) is a prevalent debilitating condition that imposes a tremendous and growing burden on the healthcare system, affected individuals, and socioeconomic factors.^1^ Currently, it affects 250 million individuals globally and ranks as the second most common physical disability in our country. Various factors such as aging, gender, obesity, and trauma may contribute to the pathogenesis of OA, its main symptoms are articular cartilage degradation, synovial inflammation, osteophyte development, and subchondral bone remodeling.^2^ In the past few years, increasing evidence supports that cholesterol is a risk factor for initiating OA. Increasing animal studies have been devoted to reveal the role of cholesterol in the OA pathogenesis. Some of these studies indicated that feeding mice models (and other animals) of OA a high‐cholesterol diet (HCD) resulted in promoted OA progression and that increased cholesterol uptake in OA chondrocytes in these mice.^3^, ^4^, ^5^ In addition, other studies have found statins (cholesterol‐lowering drugs) to reduce cartilage damage in mouse models of OA.^6^, ^7^ However, the relationship between cholesterol and OA has been the subject of considerable controversy in clinical studies. Our previous study as well as quite a few clinical studies supports a correlation between high serum cholesterol levels and OA.^8^, ^9^, ^10^ In the other side, some clinical investigations have reported opposite results, concluding that there is no significant correlation between cholesterol levels and OA.^11^, ^12^ Considering the fact that high fat intake and an inactive lifestyle are major health problems in today's modern society, as well as the controversy that still exists in clinical research. Further exploration of the specific mechanisms between cholesterol and OA would be valuable. Choi et al.’s research showed that the CH25H‐CYP7B1‐RORα axis of cholesterol metabolism plays an essential role in OA progression.^5^ Moreover, Cao et al. found that cholesterol‐induced reduction in LRP3 has been linked to OA cartilage degradation.^13^ Nevertheless, the role of cholesterol in molecular mechanisms of OA pathogenesis has not been thoroughly investigated.

Circular RNAs (circRNAs) are a novel type of noncoding RNA formed by covalently closed continuous loops without a terminal 5 ‘cap and 3′ polyadenylate tail.^14^ Recent publications have highlighted the involvement of circRNAs in the OA development and progression. For instance, circPDE4B has been shown to act as a scaffold for RIC8A and MID1, promoting articular cartilage repair and preventing deterioration.^15^ Additionally, circPDE4D has been found to mitigate OA by modulating FGF18 and miR‐103a‐3p.^16^ However, the relationship between circRNAs and cholesterol metabolism in OA remains unexplored.

Vimentin, the primary component of intermediate filament protein, is involved in the epithelial–mesenchymal transformation, promotes metastasis through cytoskeleton reprogramming, and functions as a stem cell scaffold.^17^ A previous study showed that the Vimentin expression in chondrocytes of the OA rat model was reduced by 20%.^18^ Type II collagen (COL2A1) and Aggrecan expression are decreased, while matrix metalloproteinases (MMPs) and a disintegrin and metalloproteinase with thrombospondin motif (ADAMTS) protein expression are increased in chondrocyte extracellular matrix (ECM) degradation, which is an important and significant characteristic of OA cartilage.^19^, ^20^, ^21^ Vimentin downregulation has been reported to reduce expression of the Aggrecan and COL2A1 genes in chondrocytes and lead to ECM degradation.^22^ Posttranslational modifications, such as ubiquitination, acetylation, and phosphorylation, can affect the stability and functionality of Vimentin.^23^, ^24^

In our study, we intend to analyse whether the total cholesterol (TC) level in synovial fluid of OA patients could serve as a potential diagnostic marker for OA. In addition, we also investigated the function role of the cholesterol/circARPC1B/Vimentin axis in OA progression and provided a latent therapeutic target for the OA treatment.

## 2 MATERIALS AND METHODS

### 2.1 Human synovial fluid and cartilage sample collection

Human synovial fluid and cartilage samples were collected and categorised according to protocols approved by the ethics committee of the First Affiliated Hospital of Zhejiang University School of Medicine. Obtained informed consent from all patients. OA was diagnosed by experienced orthopaedic surgeons and rheumatologists based on clinical information related to the American College of Rheumatology classification criteria.^25^ Due to the patient's medication status being considered an important exclusion criterion for the study, cartilage samples were strictly selected from patients who had no history of OA medication treatment (including steroid or non‐steroidal anti‐inflammatory drug treatment), rheumatoid arthritis, infectious arthritis, or severe underlying disease within 3 years of total knee arthroplasty. The control cartilage tissue was taken from knee fracture patients without a history of OA (*n* = 7). Pathological cartilage tissues were obtained from 67 individuals with a history of OA who underwent total knee arthroplasty. The clinical information of the patients is listed in Table S1. Human cartilage specimens were evaluated using the Kellgren–Lawrence grade (KL, preoperative imaging) and the OARSI histopathology grading system (histological staining). Details of the KL and the OARSI grading system was shown in Additional file 1.

### 2.2 Cell culture

Human chondrocyte line C28/I2 was bought from Sigma–Aldrich (St. Louis, MO, USA) and HEK293T cell line (ATCC: CRL‐1573) was bought from the American Type Culture Collection (ATCC, Manassas, VA, USA). Primary mouse chondrocytes (MCs) were isolated from the tibial plateau and femoral condyle at postnatal day 5 by 0.2% collagenase digestion (Sigma–Aldrich).^26^ Chondrocytes and HEK293T cells were cultured in the Dulbecco's modified Eagle's medium (DMEM, BasalMedia, Shanghai, China) supplemented with 10% Fetal bovine serum (FBS, Procell, Wuhan, China), 1% penicillin/streptomycin (1%, Procell) at 37°C and under 5% CO_2_ incubation.

### 2.3 Cholesterol assay

TC levels in synovial fluid, serum, cells and cartilages were detected using a Total Cholesterol Assay Kit (Solarbio, Beijing, China) according to the manufacturer's instruction and the detailed method was shown in Additional file 1. Nitrobenzoxadiaz (NBD)‐cholesterol (Biofound Biotechnology Co, Beijing, China), a fluorescent cholesterol analogue, was used to assess the cholesterol absorption level of cells. Briefly, chondrocytes were cultured for 24 h in serum‐free DMEM containing 5 μg/mL NBD‐cholesterol with or without 10 μg/mL IL‐1β (Peprotech, NJ, USA), 50 μg/ml Tumor necrosis factor α (TNFα, Peprotech), or hydrogen peroxide. After the treatment, the cells were fixed in 4% paraformaldehyde (PFA) for 15 min and stained with DAPI (Sigma–Aldrich) for 20 min. The cells were observed using a fluorescence microscope (NIKON TE2000, Nikon Corporation, Japan).

### 2.4 Quantitative real‐time polymerase chain reaction

Total RNA was extracted from chondrocytes using the TRIzol Reagent (CWBIO, Beijing, China). For RNase R treatment, 2 mg of total RNA was treated with or without 3 U/mg Rnase R (Epicenter Technologies) at 37°C for 15 min. Evo M‐MLV RT premix for qPCR(Accurate Biotechnology(Hunan)Co., Ltd, ChangSha, China) was used to reverse transcribe total RNA into cDNA, which was then quantified by real‐time fluorescent quantitative PCR. Specific primers were synthesised to amplify circARPC1B, and the amplified products were detected through agarose gel electrophoresis and sequencing. For mRNA analysis, the 2X HotStart SYBR qPCR Master Mix (Bioeast Biotech Co., Ltd, Hangzhou, China) was used, and the reactions were subsequently measured using the ABI Prism 7500 Fast System (Applied Biosystems, CA, USA) following the manufacturer's protocols. The expression level of each gene was calculated using the 2 − ΔΔ*Ct* method, with β‐actin serving as the endogenous control. The method used to design the divergent primers for circRNAs PCR was shown in Addition file 1 and the primer sequences are shown in Table S2.

### 2.5 Western blotting

Protein extraction was performed by lysing primary MCs and C28/I2 cells in RIPA buffer (Beyotime Biotechnology, Shanghai, China) supplemented with protease inhibitor (Fudebio, Hangzhou, China). The protein concentration was measured using a bicinchoninic acid protein assay kit (Beyotime). Equivalent amounts of protein were separated by 10% sodium dodecyl sulfate polyacrylamide gel electrophoresis (SDS‐PAGE) and transferred to a polyvinylidene fluoride (PVDF) membrane (EMD Millipore). The membranes were blocked and then incubated with primary antibodies for 12 h at 4°C, then incubated with corresponding secondary antibodies (Proteintech, Wuhan, China) for one hour. β‐actin was used as the internal standard, and the relative grey level of proteins was calculated using ImageJ software (NIH, Bethesda, MD, USA). The antibodies used were as follows: anti‐β‐actin antibody (1:2000, Proteintech, 66009‐1‐Ig), anti‐LOX1 antibody (1:1000, Proteintech, 11837‐1‐AP), anti‐Aggrecan antibody (1:1000, Sigma–Aldrich, c8035), anti‐COL2A1 antibody (1:1000, abcam, ab34712), anti‐MMP3 (1:1000, Proteintech, 17873‐1‐AP), anti‐MMP13 (1:1000, abcam, ab39012), anti‐ADAMTS4 (1:1000, Proteintech, 11865‐1AP), anti‐ADAMTS5 (1:1000, abcam, ab41037), anti‐Vimentin (1:1000, Cell Signaling Technology, 5741), anti‐FLAG (1:1000, abcam, ab205606), and anti‐SYVN1 (1:1000, Proteintech, 67488‐1‐Ig).

### 2.6 Immunofluorescence

Primary MCs and C28/I2 cells were fixed in 4% PFA for 20 min, permeabilised with 0.1% Triton X‐100 for 30 min, and then blocked with 5% Bovine serum albumin (BSA) at room temperature (RT) for 30 min. The cells were incubated with the following primary antibodies: anti‐LOX1 antibody (1:200, Proteintech, 11837‐1‐AP), anti‐Aggrecan antibody (1:200, Sigma–Aldrich, c8035), anti‐COL2A1 antibody (1:200, Abcam, ab34712), anti‐Vimentin (1:200, Cell Signaling Technology, 5741), and anti‐SYVN1 (1:200, Proteintech, 67488‐1‐Ig). After rinsing with phosphate buffer saline (PBS), the cells were incubated with fluorescent secondary antibodies at RT for 1 h. Subsequently, the cells were rinsed with PBS and stained with DAPI at RT for 20 min. Finally, the target proteins were observed using a fluorescence microscope (NIKON TE2000, Nikon Corporation, Japan).

### 2.7 OA model in mice

Experimental OA induced by medial meniscus destabilisation (DMM) in 12‐week‐old male mice following anaesthesia with 75 mg/kg ketamine and 10 mg/kg xylazine. The specific steps of DMM surgery refer to the previous literature.^27^ Sham‐operated mice were used as the control group. The mice were randomly divided into four groups, each containing 6 mice: SHAM + NC vector, SHAM + circARPC1B, DMM + NC vector, and DMM + circARPC1B groups. DMM surgery was performed on the right knee of the mice as described in a previous article.^27^ One week after the surgery, 10 μL of AAV (approximately 1 × 10^12^ μg/mL) (HanBio, Shanghai, China) was injected into the knee joints using 10 μL microsyringe with 34 G needle (Hamilton, Switzerland). After 8 weeks, the mice were sacrificed, and the articular cartilage of their knees were collected for histological and gene expression analysis. Detailed method of generation of AAV expressing circARPC1B was shown in Additional file 1.

### 2.8 High cholesterol diet in mice

According to a previous article,^5^ 6‐week‐old male mice were fed with the AIN‐76A diet (Trophic Animal Feed High‐Tech Co., Ltd, China) as their regular diet (RD). For the HCD diet, the mice were fed with AIN‐76A diet supplemented with 2% cholesterol. DMM surgery was performed on the right knee of the mice after 6 weeks of being on the RD or HCD. These mice continued to be fed with RD or HCD and were sacrificed 6 weeks after surgery for histological and gene expression analysis.

### 2.9 Cell counting kit‐8 assay

The Cell counting kit‐8 (CCK‐8) reagent (Dojindo Molecular Technology, Kumamoto, Japan) was used to evaluate Cholesterol's cytotoxicity on chondrocytes and detailed method was shown in Additional file 1.

### 2.10 Micromass culture

To evaluate the cartilage differentiation and glycosaminoglycan (GAG) deposition, 2×10^5^ chondrocytes were suspended in 10 μL of the conventional medium and seeded as high‐density cell masses in the center of a 24‐well plate. After 1 h, 0.5 mL medium was supplemented to each well and refreshed every other day. After 7 days, cells were fixed and stained with 1% Alcian blue (Solarbio) for 2 h. Finally, the relative level of proteoglycans was determined by measuring the intensity of Alcian blue staining using ImageJ.

### 2.11 RNA isolation and library construction for RNA‐seq

To identify circular RNAs affected by cholesterol in chondrocytes, C28/I2 cells (*n* = 3 in each group) were seeded in 10 cm cell culture dishes and treated with or without 10 μg/mL cholesterol for 48 h (Selleck, Houston, TX, USA). Total RNA was then extracted using the TRIzol kit. The quantity of the library was determined using the Agilent 2100 biological analyser (Paloalto, CA, USA) and ABI StepOnePlus RT‐PCR system (Thermo Fisher). Transcriptome sequencing was performed on six cell specimens using an Illumina novaseq 6000 platform from Novo Gene Co., Ltd. (Tianjin, China). Differential expression transcripts were identified based on the following criteria: |log2 (fold change) | > 0 and *P*‐value < 0.05. Sequences of circRNAs were retrieved from the RNA‐seq analysis and compared in circbase, circbank, and circAtlas.^28^, ^29^, ^30^ The detailed method was described in Additional file 1 and sequences of whole list of circRNAs were shown in Additional file 2.

### 2.12 RNA fluorescent in situ hybridisation

CY3‐labeled circARPC1B probes were synthesised by HaoKeBio (Hangzhou, China). The probe signal was detected using fluorescent in situ hybridisation (FISH) Kit (RiboBio, Guangzhou, China), and the nucleus was stained with DAPI. Images were observed using a fluorescence microscope (NIKON TE2000, Nikon Corporation, Minato, Tokyo, Japan). The sequences of the probes are shown in Table S2. The detailed method was shown in Additional file 1.

### 2.13 RNA pull‐down assay and mass spectrometry

The RNA pull‐down assay was conducted using the RNA pull‐down (RPD) kit (BersinBio, Guangzhou, China) following the protocols. LacZ probe (control probe) and circARPC1B probe were purchased from BersinBio. The obtained protein from the RPD experiment was then subjected to enzymolysis using trypsin (Promega, Madison, Wisconsin, USA). The enzymolysis samples were analysed using the Q‐Exactive HFX mass spectrometer (Thermo Fisher). The MS data were subsequently analysed using MASCOT (Matrix Scientific, SC, USA) to acquire qualitative identification information of target protein polypeptide molecules. Detailed method was shown in Additional file 1. The probes are shown in Table S2.

### 2.14 Silver staining

Silver staining was performed using a Fast Silver Stain Kit (Beyotime), as described in the protocol. Detailed method was shown in Additional file 1.

### 2.15 Co‐immunoprecipitation

Proteasome inhibitor MG132 (20 μ M, Selleck) was added to the cell cultures for 8 h, and then the cells were lysed. Co‐immunoprecipitation (Co‐ip) assay was conducted using the Pierce Classic Magnetic IP Kit (Thermo Fisher) following the manufacturer's guidelines, with antibodies (1:100 dilution) specific for FLAG (Abcam) and Vimentin (Cell Signaling Technology). The immunoprecipitated proteins were quantified using western blotting. Detailed method was shown in Additional file 1.

### 2.16 RNA immunoprecipitation

The RNA immunoprecipitation (RIP) assay was conducted using the RIP kit (BersinBio) following the manufacturer's guidelines. The antibodies used were IgG (Cell Signaling Technology, 7074), Vimentin (Cell Signaling Technology, 5741), AGO2 (Abcam, ab32381), and FLAG (Abcam, ab205606). The amount of circARPC1B was determined by qPCR. Detailed method was shown in Additional file 1.

### 2.17 Crosslinking‐immunoprecipitation

The crosslinking‐immunoprecipitation (CLIP) assay was conducted using the CLIP kit (ruqi.bio, Guangzhou, China) following the manufacturer's guidelines. The antibodies used were IgG (Cell Signaling Technology, 7074) and Vimentin (Cell Signaling Technology, 5741). The amount of circARPC1B was determined by qPCR. The detailed method was shown in Additional file 1.

### 2.18 Plasmids and small interfering RNA

The human linear circARPC1B sequence was synthesised and subcloned into pEX‐3‐circRNA (pGCMV/MCS/Neo, Gene Pharma, Shanghai, China). For the RIP assay, human VIM cDNA was synthesised and its truncations were sub‐cloned into the TK‐PCDH‐copGFP‐T2A‐FLAG‐Puro lentivirus overexpression vector (Tsingke, Beijing, China). Human lentivirus‐small hairpin RNA (sh)‐circARPC1B and lentivirus‐sh‐VIM were purchased from Tsingke and the backbone of shRNA is plko.1‐copGFP‐PURO. Lentivirus was ultracentrifuged, concentrated, validated, and added to the culture medium in the presence of 5μL/mL polybrene (gene pharma). After infection, cells were screened with puromycin (GIBCO, NY, USA). Small interfering RNAs (SiRNAs) were purchased from Tsingke. The detailed methods are shown in Additional file 1 and the sequences of all plasmids and siRNAs are shown in Table S2.

### 2.19 Filipin staining for cholesterol assay

TC in cartilage was detected using the Frozen Section Total Cholesterol Filipin Fluorescence Staining Kit (HPBIO‐JM4782, Hepengbio, China) following the manufacturer's instructions. The detailed method was shown in Additional file 1.

### 2.20 Histological analyses and immunohistochemistry

Cartilage samples were fixed in 4% PFA and decalcified for paraffin embedding. The sections were stained with Safranin‐O/Fast green (Solarbio) or Alcian blue, and the severity of OA was quantified by two independent blinded observers using the OARSI grade. The detailed method was shown in Additional file 1.

For immunohistochemistry (IHC), the sections were incubated with primary antibodies for 12 h at 4°C. The primary antibodies used were as follows: Aggrecan (1:400, Proteintech, 13880‐1‐AP); Vimentin (1:3000, Proteintech, 10366‐1‐AP). Then, the sections were incubated with secondary antibodies at RT for 1 h. All positively stained cells along the joint surface of each sample were counted in the tibial plateau region. Quantitative analysis was performed using Image‐Pro Plus software (MEDIA CYBERNETICS).

### 2.21 Micro‐CT analysis

The knee joints of mice were dissected and fixed with 4% PFA. The experimental parameters are: X‐ray energy of 50 kV and current of 200 μA. The aluminium filter has a size of 0.5 mm, a rotation step of 0.4° and an isometric resolution of 9 μm. 3D reconstructions of CT scans, including transverse, coronal and sagittal sections, were generated.

### 2.22 Molecular docking

The multimer model in Alphafold v2.3.2 was used to predict Vimentin and SYVN1 for X‐polymer structure prediction, all parameters used were default parameters, the database versions used were as follows: uniport, uniref90 for 2023‐03‐01, pdb_mmcif, pdb_seqres for 2023‐03 −03, and the rest of the database versions are the default versions.^31^ Use Pymol v2.5.0 to obtain a picture of the multimer obtained from the prediction. The predicted multimer structures were analysed using PRODIGY v2.0 to obtain the binding affinity and dissociation constants of the multimers formed between Vimentin and SYVN1.^32^, ^33^, ^34^ The predicted multimer structures were analysed using Protein Interactions Calculator to obtain the interactions formed between Vimentin and SYVN1.^35^

### 2.23 Statistical analysis

The data were evaluated using GraphPad Prism 9 or MedCalc V19.0.4. Data were presented as means ± SEM. Statistical comparisons between two groups using the D'Agostino and Pearson test, *F*‐test for homogeneity of variance, and *t*‐test. Multiple comparisons were conducted using the D'Agostino and Pearson test, *F*‐test, and one‐way analysis of variance (ANOVA) with the Brown–Forsythe test. Independent sample bivariate comparisons were conducted using the two‐way ANOVA with the Sidak test. Cholesterol levels in synovial fluid and serum were analysed using the Kolmogorov–Smirnov test and nonparametric test (Kruskal–Wallis test) with Dunn's multiple comparisons tests. Cholesterol clinical correlations were examined using Pearson correlation analysis. A receiver operating characteristic curve (ROC curve) was constructed with the prediction probability of OA as an alternative marker. The area under the ROC curve (95% confidence interval) was used as the accuracy index to evaluate the diagnostic efficacy of cholesterol. As shown in the figure below, it has statistical significance (*P < .05, **P < .01, ***P < .001, ****P < .0001).

## 3 RESULTS

### 3.1 Cholesterol in synovial fluid has shown potential as a diagnostic marker for osteoarthritis and osteoarthritis chondrocytes exhibit elevated cholesterol levels due to enhanced uptake

**FIGURE 1:**
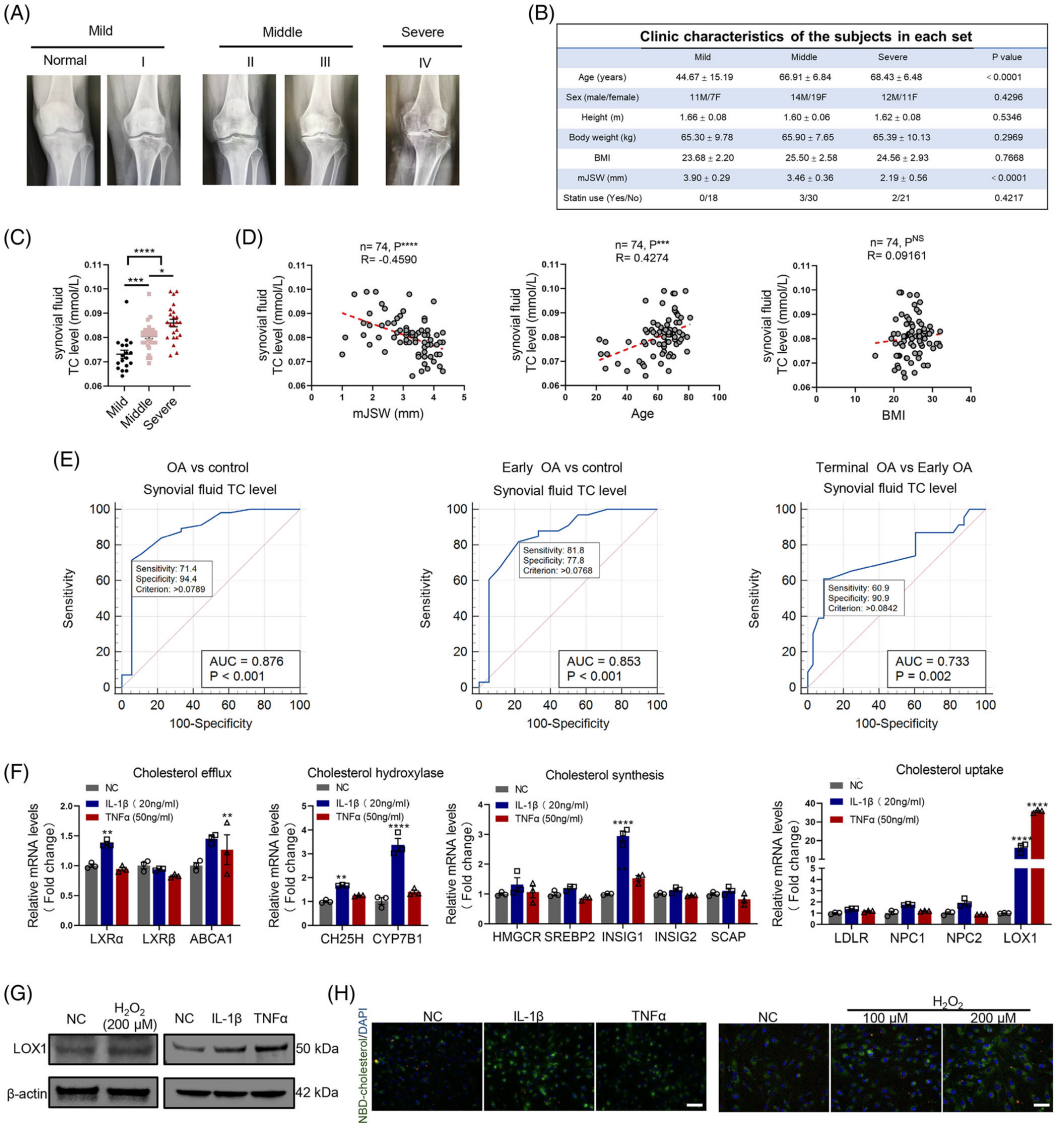
Cholesterol in synovial fluid is predictive for OA diagnosis and OA chondrocytes showed elevated cholesterol level through enhanced uptake. (A) Representative knee radiographs of patients in different OA stages. Patients were divided into five stages according to the tibiofemoral Kellgren–Lawrence (KL) score. Mild OA includes normal and stage I, and middle OA includes stages II and III with stage IV defined as severe OA; (B) clinical data of patients including age, sex, height, body weight, BMI, minimal medial joint space width (mJSW) and history of statin use. (C) The level of total cholesterol in synovial fluid of patients with mild, middle and severe OA. (D) Pearson correlation analysis between total cholesterol level in synovial fluid and mJSW of patients with OA, age and BMI. (E) Receiver operating characteristic (ROC) curve for total cholesterol in synovial fluid discriminating control and OA, control and early OA, early OA and terminal OA. Control, early OA, and terminal OA represent patients with mild OA, middle OA, and severe OA, respectively, according to the KL grades. AUC, area under the ROC curve. (F) mRNA levels of proteins involved in cholesterol efflux, hydroxylase, synthesis and uptake in C28/I2 cells treated with IL‐1β or TNFα for 48 h (*n* = 3). (G) LOX1 protein level in C28/I2 cells treated with hydrogen peroxide, IL‐1β or TNFα for 48 h (*n* = 3). (H) Representative images (*n* = 3) of NBD‐cholesterol uptake in C28/I2 cells with IL‐1β, TNFα or hydrogen peroxide for 48 h. Scale bar, 200 μM. The results were presented as mean ± SEM. *P<.05, **P<.01, ***P<.005 and ****P<.001 as compared with the control group.

We collected cartilage samples and synovial fluid from OA patients who underwent total knee arthroplasty (*n* = 67) and patients with knee fractures but no history of OA (*n* = 7). These samples were divided into three stages (mild, middle, and severe) based on KL grades (Figure 1A). Among the three stages, there were no obvious differences in gender, height, weight, body mass index (BMI) and statin use history. However, age varied among the patients, which is expected since OA is an age‐related disease (Figure 1B). Next, we evaluated the TC levels in the patient's serum and synovial fluid. Interestingly, TC levels were remarkably higher in the serum and synovial fluid of middle and severe OA patients compared to individuals with mild disease. In addition, patients with severe OA had considerably higher synovial fluid TC levels compared to those with middle‐stage OA and no such difference was observed in serum TC levels (Figure 1C and S1A), indicating a possible relationship between synovial fluid TC levels and OA progression. Pearson correlation analysis showed a negative correlation between synovial fluid/serum TC levels and the minimal medial joint space width (mJSW), which is an important index for evaluating OA through joint space narrowing on X‐rays. Additionally, synovial fluid/serum TC levels are positively correlated with age, but not with BMI (Figures 1D and S1B). Given the strong association between cholesterol and OA, further diagnostic testing was necessary. To assess the diagnostic potential of cholesterol, we constructed the ROC curve. The results indicated that synovial fluid TC levels can effectively differentiate patients with OA from generally healthy individuals. Furthermore, it showed promise in distinguishing between terminal and early‐stage OA with good sensitivity and specificity. Nevertheless, although serum TC level can also differentiate patients with OA from generally healthy individuals, it is not effective in identifying terminal and early‐stage OA (Figures 1E and S1C). Intracellular cholesterol homeostasis is controlled by cholesterol synthesis, influx, efflux and metabolism.^36^ In our study, we analysed the mRNA expression of cholesterol metabolism‐related genes in OA chondrocytes and discovered that the expression of the cholesterol uptake‐related gene lectin‐type oxidised low‐density lipoprotein receptor 1 (LOX1) exhibited the most significant increase (Figure 1F). Additionally, through western blotting and immunofluorescence (IF) assays, we confirmed that the expression level of LOX1 protein in chondrocytes increased under the influence of inflammatory factors and oxidative stress (Figures 1G and S1D,E). Oxidative stress, like inflammatory factors, plays a critical role in the OA progression.^37^, ^38^ Finally, we demonstrated that the uptake of cholesterol by chondrocytes increases under the stimulation of inflammation and oxidative stress, as demonstrated using NBD‐cholesterol, a fluorescent cholesterol analogue. (Figure 1H). These findings indicate that synovial fluid TC levels could serve as a latent marker for diagnosing OA, as OA‐related catabolic signals stimulate LOX1‐mediated cholesterol uptake in chondrocytes.

### 3.2 High cholesterol suppresses chondrocytes viability and aggravates extracellular matrix degradation

**FIGURE 2:**
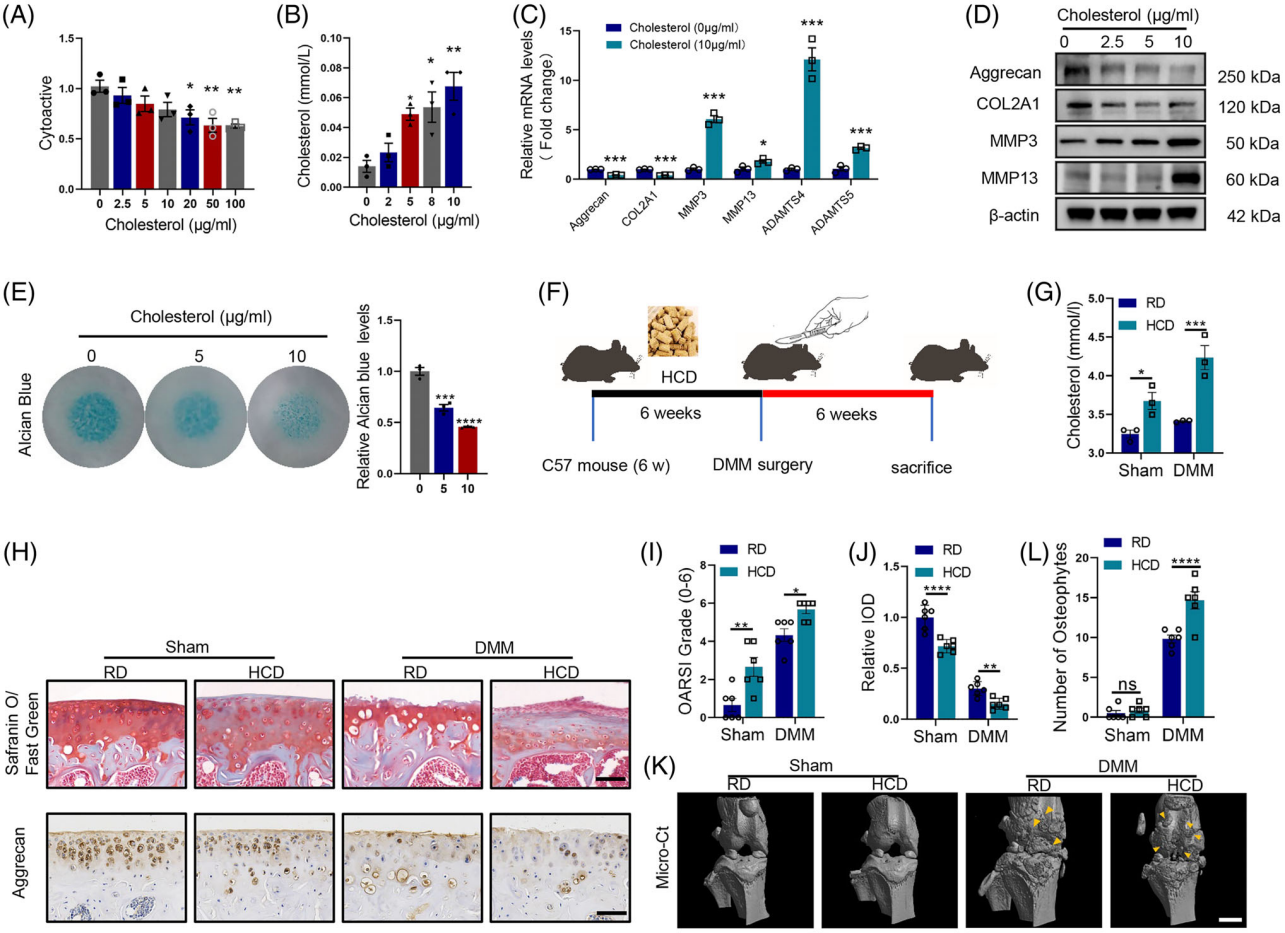
High cholesterol suppresses chondrocytes viability and aggravates ECM degradation. (A) Cholesterol impact on C28/I2 cells viability was detected by CCK‐8 assay at 48 h (*n* = 3). (B) Total cholesterol level in C28/I2 cells treated with 0, 2, 5, 8, 10 μg/mL cholesterol (*n* = 3). (C) The mRNA expression level of COL2A1, Aggrecan, MMP3, MMP13, ADAMTS4, and ADAMTS5 in C28/I2 cells treated with cholesterol (*n* = 3). (D) COL2A1, Aggrecan, MMP3, MMP13, ADAMTS4, and ADAMTS5 protein levels in C28/I2 cells treated with 0, 2.5, 5, 10 μg/mL cholesterol for 48 h (*n* = 3). (E) Micromass culture and quantification of C28/I2 cells treated with 0, 5, 10 μg/mL cholesterol for 7 days (*n* = 3). (F) Flowchart of the high cholesterol diet (HCD) feeding and DMM surgery in mice. (G) The serum TC of HCD mice compared with RD mice in Sham and DMM group (*n* = 6). (H) Representative images (*n* = 6) of safranin O/fast green and aggrecan labelled IHC staining of cartilage in four study group. Scale bar, 50 μm. (I) OARSI grade used for evaluation of the cartilage degradation in the four groups (*n* = 6). (J) Quantitative analysis of Aggrecan expression in the cartilage with IHC in the four group. (*n* = 6). (K) The representative micro‐computerised tomography images (*n* = 6) of knee joints and osteophytes (yellow arrow). Scale bars, 1 mm. (L) Quantification of osteophytes number in the four groups (*n* = 6). The results were presented as mean ± SEM. *P<.05, **P<.01, ***P<.005 and ****P<.001 as compared with the control group.

Considering the increased TC levels observed in OA chondrocytes, which is primarily attributed to elevated cholesterol uptake, we conducted experiments to investigate the effects of high cholesterol on chondrocytes. The CCK8 assay was used to measure chondrocyte proliferation, and the results revealed a significant inhibition of proliferative activity in both C28/I2 cells and MCs when the cholesterol concentration exceeded 10 μg/mL (Figures 2A and S2B). Furthermore, we confirmed that the supplement of cholesterol to the culture medium indeed increased the TC level in chondrocytes (Figures 2B and S2C). High cholesterol levels had a suppressive effect on the expression of Aggrecan and COL2A1, crucial components of the ECM. Conversely, the expression of matrix catabolic enzymes MMPs and ADAMTS were predominantly increased at the mRNA level in C28/I2 cells and MCs (Figures 2C and S2D). These findings were consistent with the outcomes observed at the protein level (Figures 2D and S2A,E,F). Additionally, Alcian blue of C28/I2 cells and MCs indicated that high cholesterol caused dose‐dependent destruction of the chondrocyte ECM (Figures 2E and S2G). To further highlight the role of cholesterol in the OA development, we investigated the effect of an HCD compared to an RD on DMM‐induced OA in mice (Figure 2F). We measured the serum TC levels in the mice and observed an increase in serum TC levels in mice fed with HCD (Figure 2G). In addition, we also measured the cartilage TC and fluorescence intensity of Filipin staining at the tibial plateau in mice and found that HCD exhibited increased chondrocytes TC levels (Figure S2H,I). Intriguingly, we also found that the cholesterol content in the surface cartilage of the knee joint of mice on both RD and HCD significantly increased after DMM surgery, which may be related to the previous conclusion that OA promotes the cholesterol uptake of chondrocytes (Figures S2I and S1F–H). In both the DMM and Sham groups, mice fed HCD exhibited more cartilage degradation compared to those on the RD (Figure 2H–J). Furthermore, after DMM surgery, mice in the HCD group developed more osteophytes than mice in the RD group, providing evidence that HCD can induce articular degeneration and exacerbate the severity of post‐traumatic OA (Figure 2K,L). Overall, our findings suggest that elevated cholesterol accelerates chondrocyte deterioration, both in vitro and in vivo.

### 3.3 High cholesterol inhibits circARPC1B expression in chondrocytes

**FIGURE 3:**
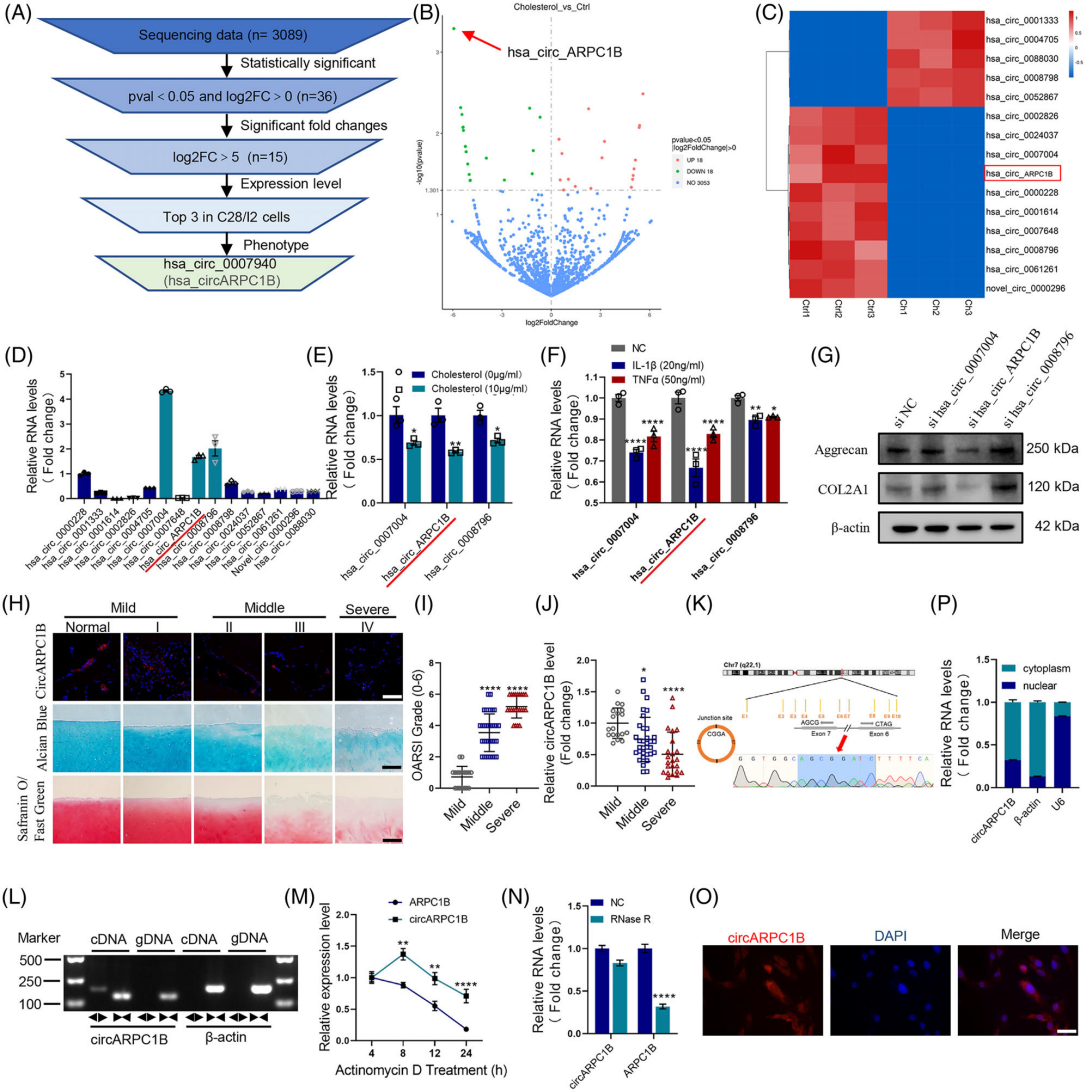
High cholesterol inhibits the circARPC1B expression in chondrocytes. (A) The flowchart illustrating the selecting processes of circARPC1B based on the sequencing data. (B) Volcano plot of 3089 circRNA were identified from sequencing data. (C) Heatmap of 15 circRNA (pval<0.05 and log2FC>5). (D) 15 circRNA expression levels in C28/I2 cells (*n* = 3). (E) hsa_circ_0007004, hsa_circ_ARPC1B and hsa_circ_0008796 expression levels in C28/I2 cells treated with 10 μg/mL cholesterol (*n* = 3). (F) hsa_circ_0007004, hsa_circ_ARPC1B and hsa_circ_0008796 expression levels in C28/I2 cells treated with IL‐1β or TNFα (*n* = 3). (G) Aggrecan and COL2A1 protein level in C28/I2 cells infected with hsa_circ_0007004, hsa_circ_ARPC1B or hsa_circ_0008796 siRNA (*n* = 3). (H) Representative images of RNA fluorescence in situ hybridisation (FISH), Safranin‐O/Fast green staining and Alcian blue staining in human cartilage tissues from stage 0 (S0) to stage 4 (S4). Scale bar, 100 mm. (I) OARSI grade used for evaluation of the cartilage degradation of patients with mild, middle, and severe OA (*n* = 74). (J) The expression of circARPC1B in human cartilage specimens of patients with mild, middle, and severe OA (*n* = 74). (K) Schematic illustration demonstrating the circularisation of ARPC1B exon 6−7 to form circARPC1B. The presence of circARPC1B was validated by RT‐PCR followed by Sanger sequencing. The red arrow represents “head‐to‐tail” circARPC1B splicing sites. (L) The presence of circARPC1B in C28/I2 cells was validated by RT‐qPCR. Divergent primers amplified circARPC1B from cDNA but not from genomic DNA; β‐actin served as the negative control. (M) The levels of circARPC1B and ARPC1B in C28/I2 cells treated with actinomycin D at the indicated time points were detected by RT‐qPCR (*n* = 3). (N) The expression of circARPC1B and linear ARPC1B mRNA in C28/I2 cells treated with or without RNase R was detected by qRT‐PCR (*n* = 3). (O) Representative images of FISH staining for circARPC1B localisation in C28/I2 cells. Scale bars, 50 μm. (P) Expression of circARPC1B assessed by RT‐qPCR in the nuclear and cytoplasmic fractions (*n* = 3). The results were presented as mean ± SEM. *P<.05, **P<.01, ***P<.005 and ****P<.001 as compared with the control group.

To investigate the dysregulated circRNAs in cholesterol‐treated chondrocytes, we performed RNA‐seq analyses on chondrocytes treated with or without cholesterol (*n* = 3, Figure 3A) and deposited the RNA‐seq raw data in GEO repository (GSE241126). Significant differences between the two groups were determined using a ≥ log2‐fold change and *P*‐value<.05. A total of 3 089 circRNAs were detected from the sequencing data, and 36 circRNAs transcripts were differentially expressed (Figure 3B). The whole list of circRNAs and their expression data are shown in Additional file 5. Among these, we selected 15 circular RNAs with significant upregulation or downregulation trends for further research (Figure 3C) and Sanger sequencing data showing the backspliced junction site for all the tested circRNAs (Figure S3A‐O). Next, we selected three circRNAs with the highest baseline expression values in chondrocytes (Figure 3D), and their expression was further validated using qRT‐PCR. qRT‐PCR confirmed that the three circRNAs expression decreased under the stimulation of high cholesterol and inflammatory factors, which was consistent with the sequencing data (Figure 3E,F). To verify the association between these three circRNAs and OA, we suppressed their expression in chondrocytes and found that only the inhibition of circARPC1B (hsa_circ_0007940) reduced COL2A1 and Aggrecan protein expression (Figure 3G). Furthermore, negative correlation between circARPC1B expression in human knee joints and the severity of OA was founded (Figure 3H–J). By comparing the circARPC1B sequence with the ARPC1B mRNA sequence, we confirmed that circARPC1B was a looped structure consisting of exons 6 and 7 from its parental gene. This head‐to‐tail splicing was determined by Sanger sequencing of the PCR product (Figure 3K). The expression of mARPC1B did not change in chondrocytes treated with cholesterol (Figure S3P). Furthermore, we isolated circARPC1B cDNA and gDNA from C28/I2 cells for nucleic acid electrophoresis detection. Convergent and specific divergent primers were synthesised to amplify ARPC1B. Notably, only the divergent primers for circARPC1B, but not actin, generated a PCR product, confirming the circular nature of circARPC1B (Figure 3L). Compared to linear RNA species, circARPC1B was found to be more resistant to degradation by RNase R and actinomycin D (20 μg/mL) treatment, indicating its increased stability (Figure 3M,N). The function of circRNAs is closely related to their cellular localisation. Fish and qRT‐PCR assays indicated that circARPC1B was predominantly localised in the cytoplasm (Figure 3O,P). In conclusion, our findings suggest that cholesterol‐induced cartilage degradation is associated with circARPC1B, but not linear ARPC1B transcripts.

### 3.4 circARPC1B regulates extracellular matrix metabolism in chondrocytes

**FIGURE 4:**
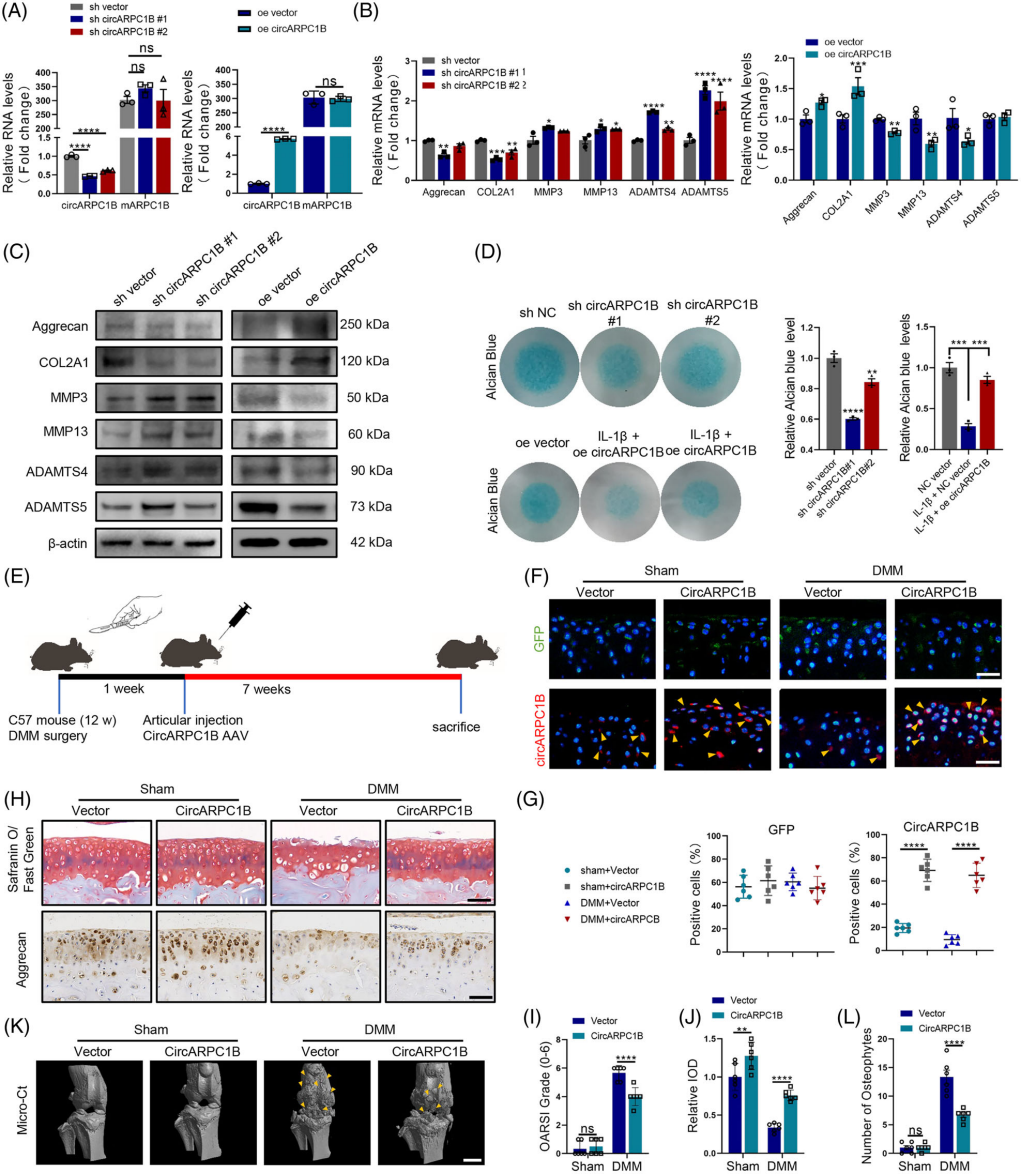
circARPC1B regulates ECM metabolism in chondrocytes. (A) circARPC1B and mARPC1B expressions in C28/I2 cells transfected with circARPC1B shRNAs or circARPC1B overexpression plasmid (*n* = 3). (B) The mRNA expression level of COL2A1, Aggrecan, MMP3, MMP13, ADAMTS4, and ADAMTS5 in C28/I2 cells after circARPC1B knockdown and overexpression (*n* = 3). (C) COL2A1, Aggrecan, MMP3, MMP13, ADAMTS4, and ADAMTS5 protein levels in C28/I2 cells after circARPC1B knockdown and overexpression (*n* = 3). (D) Micromass culture and quantification of C28/I2 cells after circARPC1B knockdown and overexpression for 7 days (*n* = 3). (E) Flowchart of the DMM surgery and circARPC1B AAV articular injection in mice. (F,G) Representative images (*n* = 6) and statistical diagrams of circARPC1B and GFP expression, Scale bar, 50 μm. (H) Representative images (*n* = 6) of safranin O/fast green and aggrecan labelled IHC staining of cartilage in four study group. Scale bar, 50 μm. (I) OARSI grade used for evaluation of the cartilage degradation in the four groups (*n* = 6). (J) Quantitative analysis of Aggrecan expression in the cartilage with IHC in the four groups (*n* = 6). (K) The representative micro–computerised tomography images (*n* = 6) of knee joints and osteophytes (yellow arrow). Scale bars, 1 mm. (L). quantification of osteophytes number in the four groups (*n* = 6). The results were presented as mean ± SEM. *P<.05, **P<.01, ***P<.005 and ****P<.001 as compared with the control group.

To explore the role of circARPC1B in ECM metabolism, we transfected C28/I2 cells with two circARPC1B shRNAs and a circARPC1B overexpression plasmid and circARPC1B expression regulation did not affect ARPC1B and β‐actin expression levels (Figures 4A and S4A). The results of Alcian blue staining in C28/I2 cells revealed that circARPC1B inhibition significantly reduced Alcian blue‐stained GAG. Conversely, circARPC1B overexpression rescued GAG loss caused by IL‐1β (Figure 4D). Moreover, MMP3, MMP13, ADAMTS4 and ADAMTS5 showed significant up‐ or down‐regulation in response to circARPC1B levels deficiency or upregulation and Aggrecan and COL2A1 exhibited primarily down‐ or up‐regulation, as confirmed by RT‐qPCR and western blot assays (Figure 4B,C). IF assays further confirmed these findings by demonstrating that changes in circARPC1B expression affected the levels of Aggrecan and COL2A1 in C28/I2 cells (Figure S4B). To investigate the functions of circARPC1B in vivo, we injected adeno‐associated virus (AAV) carrying circARPC1B into the knee joint cavity of DMM‐induced OA mice for 7 weeks (Figure 4E) and the previous literature has shown that the duration of gene expression transduction after AAV injection is at least 3 months.^39^, ^40^ This intervention significantly increased circARPC1B expression in the cartilage tissue treated with AAV‐circARPC1B (Figure 4F,G). Subsequently, we observed that the overexpression of circARPC1B partially reversed OA progression and the biochemical changes in Aggrecan induced by DMM surgery (Figure 4H–L). In conclusion, these results demonstrated that circARPC1B plays a critical role in alleviating inflammatory reactions and maintaining ECM metabolism during OA progression.

### 3.5 Vimentin interacts with circARPC1B and participates in osteoarthritis

**FIGURE 5:**
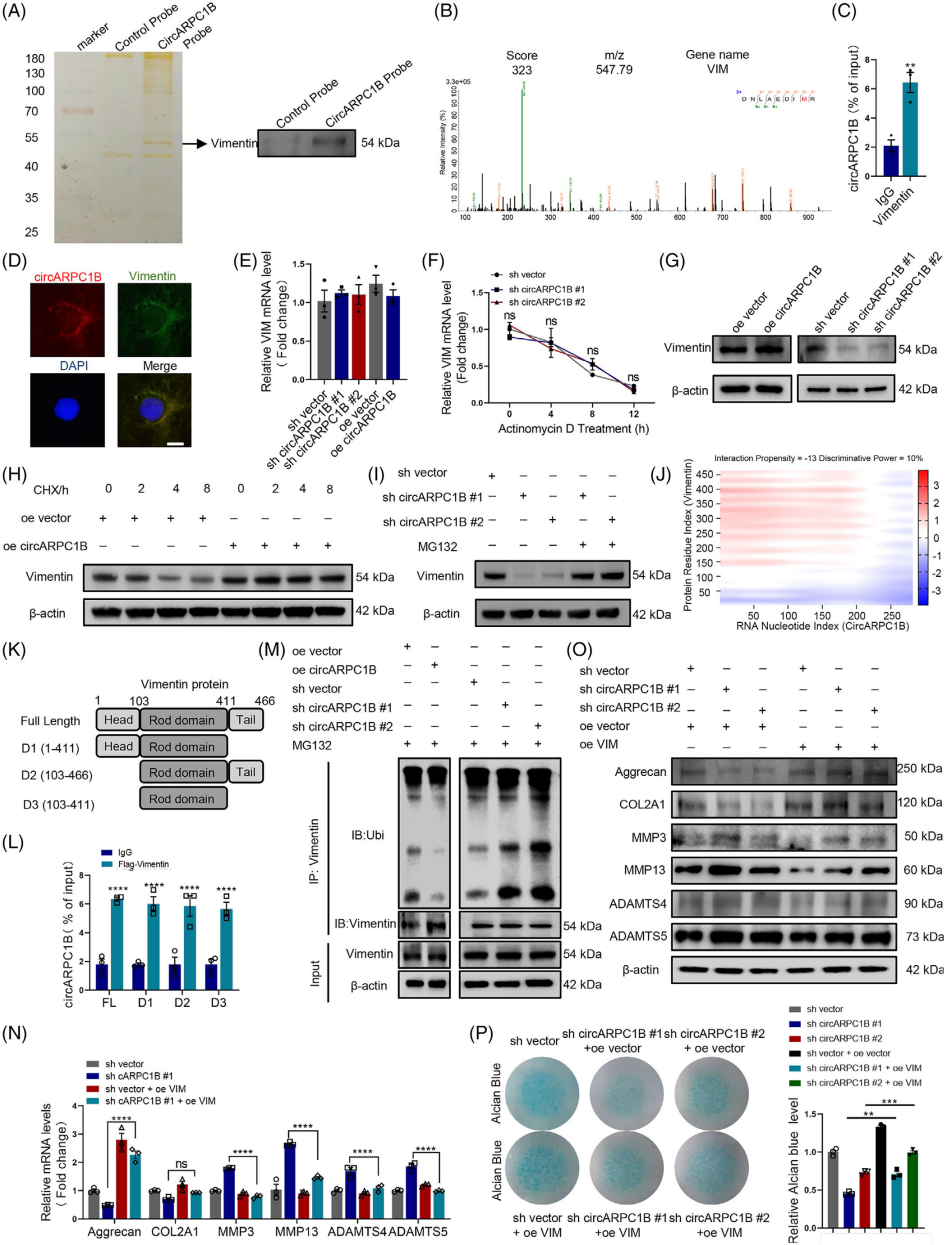
Vimentin interacts with circARPC1B and participates in OA. (A) Silver staining of proteins interacting with biotin‐circARPC1B and vimentin protein level in control and circARPC1B groups. (B) The peak map of vimentin acquired from the RNA pulldown mass spectrometry assay. (C) Vimentin‐circARPC1B interaction detected by RIP assay (*n* = 3). (D) circARPC1B and vimentin interaction in C28/I2 cells confirmed via an RNA‐protein colocalisation. Scale bar, 20 μm. mRNA levels (E), mRNA stability (F) and protein levels (G) of RIC8A after circARPC1B knockdown and overexpression (*n* = 3). (H) Vimentin protein level in C28/I2 cells treated with the transcription inhibitor CHX (200 μg/mL, *n* = 3). (I) Effect of MG132 treatment on vimentin protein level alteration mediated by circARPC1B knockdown (*n* = 3). (J) Predicted binding sites of circARPC1B and Vimentin (catRAPID graph). (K) Illustration of Vimentin and Vimentin deletion mutations. (L) Binding sequence of circARPC1B for Vimentin via RIP assay (*n* = 3). (M) IP analysis of ubiquitinated vimentin in C28/I2 cells treated with MG132. The lysates of circARPC1B overexpression or knockdown cells were treated with an anti‐vimentin antibody. (N) The effect of vimentin overexpression on COL2A1, Aggrecan, MMP3, MMP13, ADAMTS4, and ADAMTS5 mRNA levels in C28/I2 cells after circARPC1B knock‐down (*n* = 3). (O) The effect of vimentin overexpression on COL2A1, Aggrecan, MMP3, MMP13, ADAMTS4, and ADAMTS5 protein levels in C28/I2 cells after circARPC1B knock‐down (*n* = 3). (P) The effect of vimentin expression on micromass culture and quantification of C28/I2 cells after circARPC1B knockdown for 7 days (*n* = 3). The results were represented as mean ± SEM. *P < .05, **P<.01, ***P<.005 and ****P<.001 as compared with the control group.

Cytoplasm‐localised circRNAs have been implicated in translational regulation through their roles as ceRNAs, coding RNAs or protein decoys.^41^ AGO2 RIP assay revealed that circARPC1B does not bind to AGO2 (Figure S5A). Additionally, bioinformatics analysis using circRNADb software indicated that circARPC1B contains an open reading frame (ORF) fragment, but the likelihood of encoding a protein is relatively low (Figure S5B).^42^ To further validate this, we constructed a circARPC1B overexpressing vector with a FLAG tag and transfected it into C28/I2 cells. Western blot analysis using the FLAG antibody did not observe any specific bands and there were no bands observed near 10 Kda, indicating that circARPC1B cannot encode a protein (Figure S5C). To gain further insights into the regulatory mode of circARPC1B in C28/I2 cells, we employed RPD‐MS and identified a total of 308 proteins that interact with circARPC1B (Figure 5A). The top 20 CircARPC1B‐binding proteins identified by MS (ranked by prot_score) are listed in Table S3. Among these proteins, Vimentin had the highest prot_score protein score (Figure 5A,B). Vimentin has been previously associated with OA, although the exact relationship remains unclear.^18^, ^22^, ^43^, ^44^, ^45^ To further explore the role of Vimentin in chondrocytes, we constructed two VIM shRNA to stably decrease Vimentin mRNA and protein levels in C28/I2 cells and MCs (Figure S5D,E). Subsequently, we performed qRT‐PCR, western blotting, and IF, which demonstrated that VIM knockdown reduced the expression of anabolic biomarkers (Aggrecan and COL2A1), while increasing the expression of catabolic biomarkers (MMPS and ADAMTS) in C28/I2 cells and MCs (Figure S5F–H). Interestingly, Alcian blue staining of C28/I2 cells and MCs revealed that VIM inhibition significantly impaired the GAG content in chondrocytes (Figure S5I). Next, we confirmed the binding of Vimentin and circARPC1B through RIP assay in C28/I2 cells (Figure 5C). In addition, we further demonstrated an interaction between Vimentin and circARPC1B using CLIP assay (Figure S5J). Furthermore, RNA‐protein colocalisation analysis in C28/I2 cells verified the interaction between Vimentin and circARPC1B (Figure 5D). Additional experiments revealed that circARPC1B controls the protein level of Vimentin without affecting mRNA stability or levels (Figure 5E–G). We inhibited the production of Vimentin protein using cycloheximide (CHX) treatment and found that circARPC1B increased its stability, as evidenced by a significant difference in the half‐life of Vimentin protein between oe vector and oe circARPC1B C28/I2 cells (Figure 5H). Further evidence of circARPC1B regulation of Vimentin through proteasomal activity comes from the fact that the MG132 therapy corrected the decline in Vimentin protein caused by circARPC1B knockdown (Figure 5I). We predicted the possible binding regions of Vimentin and circARPC1B using the online network tool cat RAPID^46^ (Figure 5J). Moreover, to determine the specific domain of Vimentin that interacts with circARPC1B, we constructed three Vimentin deletion mutants with Flag tags (D1: 1−411, D2: 103−466, and D3: 103−411), following the methods described in a previous study^47^ (Figure 5K). The RIP assays revealed that both full‐length Vimentin and the three deletion mutants interacted with circARPC1B (Figure 5L), indicating that circARPC1B specifically binds to the Rod domain of Vimentin and not to the head and tail domains. Similarly, circARPC1B led to an increase in the polyubiquitination of Vimentin, whereas the knockdown of circARPC1B resulted in a decrease (Figure 5M). To confirm the association between circARPC1B and Vimentin in chondrocytes, we conducted a rescue experiment by co‐transfecting sh circARCPC1B and oe VIM (Figure S5K,L). Our findings demonstrated that Vimentin overexpression prevented chondrocyte degeneration caused by circARPC1B downregulation (Figure 5N–P). In conclusion, circARPC1B suppresses the ubiquitin‐proteasome pathway in OA, thereby post‐transcriptionally increasing the level of Vimentin protein.

### 3.6 CircARPC1B interacts with Vimentin to prevent its degradation by E3 ligase synovial enzyme 1 (SYVN1)

**FIGURE 6:**
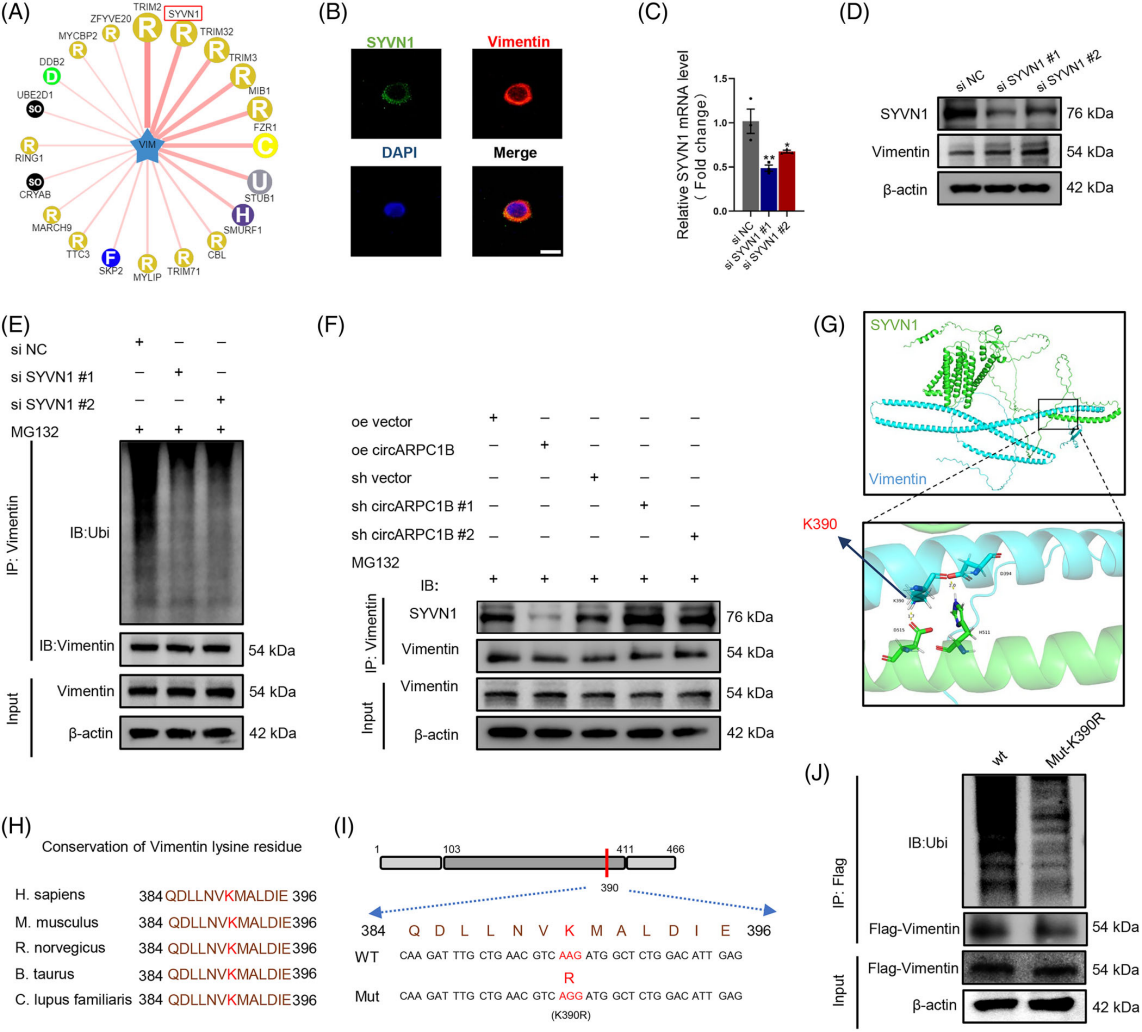
CircARPC1B interacts with vimentin to prevent its degradation by E3 ligase synovial enzyme 1 (SYVN1). (A) Prediction of potential E3 ligase of Vimentin. (B) Colocalisation in C28/I2 cells labelled with anti‐vimentin and anti‐SYVN1 by immunofluorescence, scale bar, 20 μm. (C) SYVN1 mRNA expression in C28/I2 cells transfected with SYVN1 siRNA (*n* = 3). (D) SYVN1 and vimentin protein expression in C28/I2 cells transfected with SYVN1 siRNA (*n* = 3). (E) Effect of SYVN1 knockdown on vimentin ubiquitylation (*n* = 3). (F) Effect of circARPC1B overexpression or knockdown on the interaction between vimentin and SYVN1 (*n* = 3). (G) Molecular docking predicting the interaction between SYVN1 and Vimentin. (H) Conservation of Vimentin K390 in different species. (I) K390 is located in Rod domain of Vimentin, which was mutated to arginine through “A” replaced by “G.” (J) K390R Vimentin ubiquitylation in C28/I2 cells (*n* = 3). The results were represented as mean ± SEM. *P < .05, **P<.01, ***P<.005 and ****P<.001 as compared with the control group.

The E3 ubiquitin has been reported to promote Vimentin ubiquitination and degradation in breast cancer cells.^24^ Therefore, we used the ubibrowser1.0 to predict VIM E3 ligases (Figure 6A) and top 30 E3 ligases are presented in Table S4.^48^ In this study, we chose two E3 ligases with prediction scores greater than 0.7. Surprisingly, upon the knockdown of TRIM2, Vimentin ubiquitination displayed an unexpected increase to a certain extent, instead of the anticipated decrease (Figure S6A,B). However, our results showed that SYVN1 knockout suppressed Vimentin ubiquitination degradation and IF staining of Vimentin and SYVN1 also confirmed that they colocalised with each other in C28/I2 cells (Figure 6B–E). Co‐ip assay also revealed that the binding of Vimentin and SYVN1 decreased in cells overexpressing circARPC1B compared with control cells, whereas circARPC1B knockdown induced the opposite effect (Figure 6F). The interaction model between SYVN1 and Vimentin was predicted using molecular docking. Interestingly, the prediction results revealed that SYVN1 exclusively binds to the rod domain of Vimentin, aligning with the binding region of circARPC1B to Vimentin (Figure 6G). K390 in Vimentin has the shortest hydrogen bond with 515D in SYVN1. In addition, K390 in Vimentin was highly conserved among different species, suggesting it plays an important role in Vimentin (Figure 6H). Notably, changing K390 lysine to arginine‐reduced Vimentin ubiquitination (Figure 6I,J). In conclusion, our findings suggest that circARPC1B acts as a competitive inhibitor, effectively occupying the binding sites of Vimentin that are typically targeted by SYVN1. This prevents the proteasomal degradation of Vimentin in chondrocytes, highlighting the regulatory role of circARPC1B in maintaining Vimentin stability.

### 3.7 CircARPC1B overexpression reverses high cholesterol‐induced osteoarthritis

**FIGURE 7:**
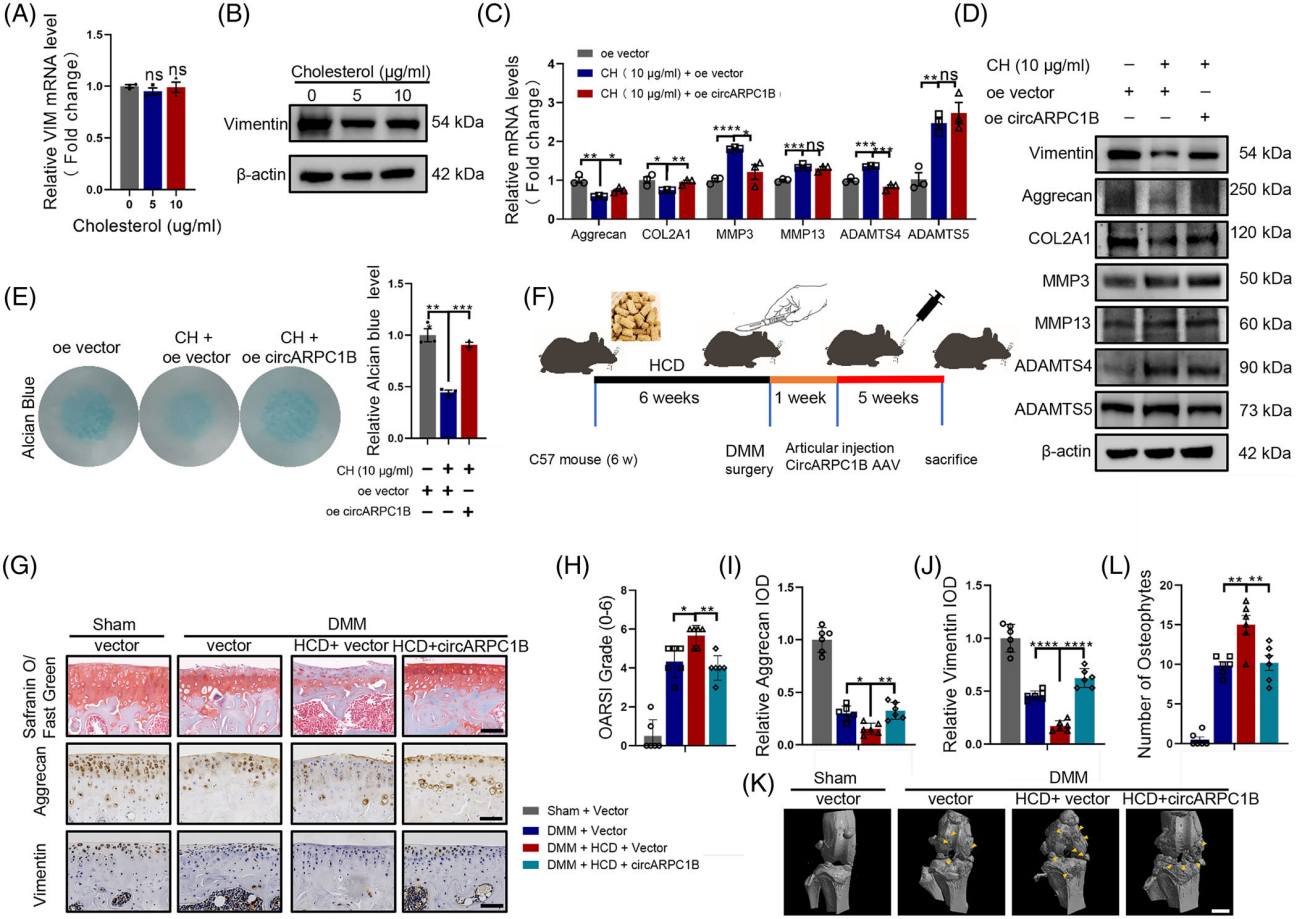
CircARPC1B overexpression reverses high cholesterol‐induced OA. (A) VIM mRNA expression in C28/I2 cells treated with 0, 5, 10 μg/mL cholesterol (*n* = 3). (B) Vimentin protein expression in C28/I2 cells treated with 0, 5, 10 μg/mL cholesterol (*n* = 3). (C) The effect of circARPC1B overexpression on COL2A1, Aggrecan, MMP3, MMP13, ADAMTS4, and ADAMTS5 mRNA expression in C28/I2 cells treated with 10 μg/mL cholesterol (*n* = 3). (D) The effect of circARPC1B overexpression on COL2A1, Aggrecan, MMP3, MMP13, ADAMTS4, and ADAMTS5 protein expression in C28/I2 cells treated with 10 μg/mL cholesterol (*n* = 3). (E) The effect of circARPC1B overexpression on micromass culture and quantification of C28/I2 cells treat with 10 μg/mL cholesterol for 7 days (*n* = 3). (F) Flowchart of the HCD feeding, DMM surgery and circARPC1B AAV articular injection in mice. (G) Representative images (*n* = 6) of safranin O/fast green, aggrecan and vimentin labelled IHC staining of cartilage in the four study group. Scale bar, 50 μm. (H) OARSI grade used for evaluation of the cartilage degradation in the four groups (*n* = 6). (I,J) Quantitative analysis of Aggrecan and vimentin expression in the cartilage with IHC in the four groups (*n* = 6). (K) The representative micro‐computerised tomography images (*n* = 6) of knee joints and osteophytes (yellow arrow). Scale bars, 1 mm. (L) Quantification of osteophytes number in the four groups (*n* = 6). The results were presented as mean ± SEM. *P<.05, **P<.01, ***P<.005 and ****P<.001 as compared with the control group.

Given the inhibitory effect of high cholesterol on circARPC1B expression in chondrocytes, our aim is to investigate whether the detrimental impact of high cholesterol on the ECM of chondrocytes is mediated through the modulation of circARPC1B. Intriguingly, high cholesterol suppressed the expression level of Vimentin protein but not its RNA level in C28/I2 and MCs (Figures 7A,B and S6C,D), which is consistent with the effect of circARPC1B on Vimentin. Moreover, circARPC1B overexpression partially reversed the cholesterol‐related OA phenotype in C28/I2 cells as demonstrated by the remarkably increased Aggrecan and COL2A1 expression and decreased MMPs and ADAMTS expression, as determined by the qPCR, western blotting and IF analyses (Figures 7C,D and S6E). Moreover, circARPC1B overexpression rescued the degradation of GAGs induced by elevated cholesterol (Figure 7E). To further elucidate the link between cholesterol and circARPC1B in OA, we conducted DMM surgery on mice that were subjected to an HCD. Additionally, 1 week following the DMM surgery, we performed intraarticular injection of AAV circARPC1B. This experimental approach aimed to provide additional insights into the relationship between cholesterol and circARPC1B in the context of OA (Figure 7F). CircARPC1B can shield chondrocytes against excessive cholesterol damage in vivo, as revealed by the OARSI scoring method, the quantity of osteophytes and the IHC assay (Figure 7G–K). Together, these findings demonstrated that circARPC1B and high cholesterol contribute to the OA pathogenesis, and Graphical Abstract details the precise mechanism.

## 4 DISCUSSION

OA is the most widespread chronic debilitating disease, mainly affecting people over 65 years old.^49^ The primary etiology of OA is attributed to an imbalance between cartilage matrix anabolism and catabolism. Despite the identification of several risk factors associated with OA, the precise mechanisms underlying cartilage matrix degradation remain elusive. Considering that OA is strongly linked to many cholesterol‐related illnesses apart from cardiovascular diseases, such as cancer, peroxisomal disorders and Alzheimer's disease, cholesterol homeostasis has received extensive research over the past few years.^50^, ^51^, ^52^ So far, the relationship between cholesterol metabolism and OA is notoriously controversial. There is quite a bit of evidence to support an interaction between cholesterol metabolism and OA. For instance, Li et al. found that OA and pain severity scores were higher in patients with hypercholesterolemia than in those with normal serum cholesterol levels.^53^ Other scholars reported that statins (cholesterol‐lowing drug) protective against OA in clinical trials.^54^, ^55^The study by Wu et al. was even more direct in finding that pravastatin significantly reduced IL‐1β induced MMP‐1 and MPP‐13 expression as well as intracellular cholesterol expression in human chondrocytes of OA patients. However, numerous studies have reported that there is no direct link between OA and cholesterol metabolism.^12^, ^56^ Our research identified an obvious and significant increase in serum and synovial fluid TC levels of patients with OA among 74 patients and synovial fluid TC levels correlated better with OA severity compared to serum TC levels. Our in vitro experiments revealed increased cholesterol uptake in OA cartilage, and the increased cholesterol further exacerbated the progression of OA. Although more samples are needed, cholesterol in synovial fluid may still be a latent biomarker for OA diagnosis. However, we still need to take into account that cholesterol includes HDL‐C, LDL‐C, IDL‐C, and VLDL‐C, and it has been reported in the literature that TC and LDL‐C levels in serum of OA patients were higher than normal, while the HDL‐C level was markedly lower.^57^ Thus, the specific type of cholesterol in synovial fluid that is strongly associated with OA progression still needs to be further explored.

Previous studies have suggested that mitochondrial dysfunction and oxidative stress may be the main mechanisms of cholesterol‐induced chondrocyte disorders.^58^ In addition, recent research on nature indicated that cholesterol and its metabolites directly activate retinoic acid‐related orphan receptor alpha in chondrocytes, which upregulates matrix‐degrading enzymes and increases the risk of OA.^5^ However, no study to date has explored the effect of cholesterol on circRNAs’ expression in chondrocytes. CircRNAs have garnered significant attention in recent years owing to their diverse biological activities, and accumulating evidence from numerous studies has highlighted their crucial role in the pathological progression of OA.^15^, ^16^, ^59^ In this study, we discovered that high cholesterol inhibited circARPC1B expression in chondrocytes and circARPC1B expression in human tibial plateau cartilage samples decreased with OA severity, implying that cholesterol may promote OA progression by inhibiting circARPC1B expression. To demonstrate this, we verified in vivo and in vitro that circARPC1B prevents cartilage ECM degradation and thus inhibits OA progression. As for the specific mechanism through which cholesterol alters circARPC1B expression in chondrocytes, it still needs to be further explored in the future.

CircRNAs bind to various proteins to form circRNA–protein complexes which are necessary for it to perform its biological function. The ability of circARPC1B to bind to Vimentin was explored using the RNA pull‐down assay. Vimentin, an important component of the cytoskeleton, plays an important role in chondrocyte hardness maintenance.^17^, ^60^ Importantly, Vimentin expression was found to be decreased in the OA model, and Vimentin abnormalities can affect the expression of anabolic biomarkers such as Aggrecan and COL2A1.^18^, ^22^ Our data validate that Vimentin downregulation disrupts cartilage ECM, but the specific mechanism by which Vimentin mediates OA requires further investigation in future. In the current study, our investigations revealed that Vimentin operates as an RNA‐binding protein for circARPC1B. This interaction between circARPC1B and Vimentin was further confirmed through techniques such as RNA‐protein colocalisation, the RIP (RNA immunoprecipitation) and CLIP assays. CircARPC1B altered the expression level of Vimentin protein but not its mRNA level, implying that circARPC1B can influence post‐translational modifications of Vimentin. As a major post‐translational modification, ubiquitylation regulates several physiological processes including chondrocytes metabolism.^15^, ^47^, ^59^ CircARPC1B knockdown increased Vimentin ubiquitination, whereas circARPC1B overexpression decreased its ubiquitination. Furthermore, rescue experiments further demonstrated that circARPC1B functions as a Vimentin stabilisation enhancer to prevent chondrocyte degradation. The relationship between Vimentin and the E3 ubiquitin ligase SYVN1 in breast cancer has previously been reported^24^ and our findings confirm that circARPC1B inhibits Vimentin degradation by blocking Vimentin‐SYVN1 binding. Surprisingly, we discovered that cholesterol inhibited Vimentin expression in chondrocytes and that circARPC1B alleviated high cholesterol‐induced OA as a Vimentin stabilisation enhancer. What cannot be ruled out is that circARPC1B may still have an effect on chondrocytes through other pathways. For example, circARPC1B may have an effect on OA by influencing miRNAs to exert non‐classical regulatory pathways,^61^, ^62^ which needs to be further explored.

## 5 CONCLUSIONS

Synovial fluid TC levels can be a potential diagnostic marker for OA. In addition, our data suggested that cholesterol‐circARPC1B‐Vimentin axis plays an essential role in OA progression and circARPC1B might be a promising therapeutic target for the OA treatment.

## CONFLICT OF INTEREST STATEMENT

The authors declare no conflicts of interest.

## FUNDING INFORMATION

By Zhejiang Provincial Natural Science Foundation of China, Grant No: LGF22H060003

## Supporting information

Supporting Information

Supporting Information

Supporting Information

Supporting Information

Supporting Information

## References

[1] Prieto‐Alhambra D , Judge A , Javaid MK , et al. Incidence and risk factors for clinically diagnosed knee, hip and hand osteoarthritis: influences of age, gender and osteoarthritis affecting other joints. Ann Rheum Dis. 2014;73(9):1659‐1664. 23744977 10.1136/annrheumdis-2013-203355 PMC3875433

[2] Hunter D , Bierma‐Zeinstra SJL . Osteoarthritis. Lancet. 2019;393(10182):1745‐1759. 31034380 10.1016/S0140-6736(19)30417-9

[3] Warmink K , Kozijn AE , Bobeldijk I , et al. High‐fat feeding primes the mouse knee joint to develop osteoarthritis and pathologic infrapatellar fat pad changes after surgically induced injury. Osteoarthr Cartil. 2020;28(5):593‐602. 10.1016/j.joca.2020.03.008 32222415

[4] Brunner AM , Henn CM , Drewniak EI , et al. High dietary fat and the development of osteoarthritis in a rabbit model. Osteoarthr Cartil. 2012;20(6):584‐592. 10.1016/j.joca.2012.02.007 22353745

[5] Choi W‐S , Lee G , Song W‐H , et al. The CH25H‐CYP7B1‐RORα axis of cholesterol metabolism regulates osteoarthritis. Nature. 2019;566(7743):254‐258. 30728500 10.1038/s41586-019-0920-1

[6] Gierman LM , Kühnast S , Koudijs A , et al. Osteoarthritis development is induced by increased dietary cholesterol and can be inhibited by atorvastatin in APOE*3Leiden.CETP mice—a translational model for atherosclerosis. Ann Rheum Dis. 2014;73(5):921‐927. 23625977 10.1136/annrheumdis-2013-203248

[7] Ali SA , Al‐Jazrawe M , Ma H , et al. Regulation of cholesterol homeostasis by hedgehog signaling in osteoarthritic cartilage. Arthritis Rheumatol. 2016;68(1):127‐137. 26315393 10.1002/art.39337 PMC4690757

[8] Gkretsi V , Simopoulou T , Tsezou A . Lipid metabolism and osteoarthritis: lessons from atherosclerosis. Prog Lipid Res. 2011;50(2):133‐140. 21115041 10.1016/j.plipres.2010.11.001

[9] Kellgren JH . Osteoarthrosis in patients and populations. Br Med J. 1961;2(5243):1‐6. 13752350 10.1136/bmj.2.5243.1 PMC1968987

[10] Hart DJ , Doyle DV , Spector TD . Association between metabolic factors and knee osteoarthritis in women: the Chingford study. J Rheumatol. 1995;22(6):1118‐1123. 7674240

[11] Xiong J , Long J , Chen X , Li Y , Song H . Dyslipidemia might be associated with an increased risk of osteoarthritis. Biomed Res Int. 2020;2020:3105248. 32149100 10.1155/2020/3105248 PMC7048911

[12] Dahaghin S , Bierma‐Zeinstra SMA , Koes BW , Hazes JMW , Pols HAP . Do metabolic factors add to the effect of overweight on hand osteoarthritis? The Rotterdam study. Ann Rheum Dis. 2007;66(7):916‐920. 17314121 10.1136/ard.2005.045724 PMC1955104

[13] Cao C , Shi Y , Zhang X , et al. Cholesterol‐induced LRP3 downregulation promotes cartilage degeneration in osteoarthritis by targeting Syndecan‐4. Nat Commun. 2022;13(1):7139. 36414669 10.1038/s41467-022-34830-4 PMC9681739

[14] Vo JN , Cieslik M , Zhang Y , et al. The landscape of circular RNA in cancer. Cell. 2019;176(4):869‐881. 30735636 10.1016/j.cell.2018.12.021 PMC6601354

[15] Shen S , Yang Y , Shen P , et al. circPDE4B prevents articular cartilage degeneration and promotes repair by acting as a scaffold for RIC8A and MID1. Ann Rheum Dis. 2021;80(9):1209‐1219. 34039624 10.1136/annrheumdis-2021-219969 PMC8372377

[16] Wu Y , Hong Z , Xu W , et al. Circular RNA circPDE4D protects against osteoarthritis by binding to miR‐103a‐3p and regulating FGF18. Mol Ther. 2021;29(1):308‐323. 33125858 10.1016/j.ymthe.2020.09.002 PMC7791010

[17] Chung B‐M , Rotty JD , Coulombe PA . Networking galore: intermediate filaments and cell migration. Curr Opin Cell Biol. 2013;25(5):600‐612. 23886476 10.1016/j.ceb.2013.06.008 PMC3780586

[18] Capín‐Gutiérrez N , Talamás‐Rohana P , González‐Robles A , Lavalle‐Montalvo C , Kourí JJH . Histopatholo, cytoskeleton disruption in chondrocytes from a rat osteoarthrosic (OA) ‐induced model: its potential role in OA pathogenesis. Histol Histopathol. 2004;19(4):1125‐1132. 15375755 10.14670/HH-19.1125

[19] Patel J , Saleh K , Burdick J , RJAb Mauck . Bioactive factors for cartilage repair and regeneration: improving delivery, retention, and activity. Acta Biomater. 2019;93:222‐238. 30711660 10.1016/j.actbio.2019.01.061 PMC6616001

[20] Mouw JK , Ou G , Weaver VM . Extracellular matrix assembly: a multiscale deconstruction. Nat Rev Mol Cell Biol. 2014;15(12):771‐785. 25370693 10.1038/nrm3902 PMC4682873

[21] Troeberg L , Nagase H . Proteases involved in cartilage matrix degradation in osteoarthritis. Biochim Biophys Acta. 2012;1824(1):133‐145. 21777704 10.1016/j.bbapap.2011.06.020 PMC3219800

[22] Blain E , Gilbert S , Hayes A , Duance VJM . bjotISfMB, disassembly of the vimentin cytoskeleton disrupts articular cartilage chondrocyte homeostasis. Matrix Biol. 2006;25(7):398‐408. 16876394 10.1016/j.matbio.2006.06.002

[23] Snider NT , Omary MB . Post‐translational modifications of intermediate filament proteins: mechanisms and functions. Nat Rev Mol Cell Biol. 2014;15(3):163‐177. 24556839 10.1038/nrm3753 PMC4079540

[24] Fan Y , Wang J , Jin W , et al. CircNR3C2 promotes HRD1‐mediated tumor‐suppressive effect via sponging miR‐513a‐3p in triple‐negative breast cancer. Mol Cancer. 2021;20(1):25. 33530981 10.1186/s12943-021-01321-x PMC7851937

[25] Altman R , Asch E , Bloch D , et al. Development of criteria for the classification and reporting of osteoarthritis. Classification of osteoarthritis of the knee. Diagnostic and Therapeutic Criteria Committee of the American Rheumatism Association. Arthritis Rheum. 1986;29(8):1039‐1049. 3741515 10.1002/art.1780290816

[26] Gosset M , Berenbaum F , Thirion S , Jacques C . Primary culture and phenotyping of murine chondrocytes. Nat Protoc. 2008;3(8):1253‐1260. 18714293 10.1038/nprot.2008.95

[27] Zhao Y , Liu B , Liu C‐j . Establishment of a surgically‐induced model in mice to investigate the protective role of progranulin in osteoarthritis. J Vis Exp. 2014(84):e50924. 24638128 10.3791/50924 PMC4131755

[28] Glažar P , Papavasileiou P , Rajewsky N . circBase: a database for circular RNAs. RNA. 2014;20(11):1666‐1670. 25234927 10.1261/rna.043687.113 PMC4201819

[29] Liu M , Wang Q , Shen J , Yang BB , Ding X . Circbank: a comprehensive database for circRNA with standard nomenclature. RNA Biol. 2019;16(7):899‐905. 31023147 10.1080/15476286.2019.1600395 PMC6546381

[30] Wu W , Ji P , Zhao F . CircAtlas: an integrated resource of one million highly accurate circular RNAs from 1070 vertebrate transcriptomes. Genome Biol. 2020;21(1):101. 32345360 10.1186/s13059-020-02018-y PMC7187532

[31] Jumper J , Evans R , Pritzel A , et al. Highly accurate protein structure prediction with AlphaFold. Nature. 2021;596(7873):583‐589. 34265844 10.1038/s41586-021-03819-2 PMC8371605

[32] Xue LC , Rodrigues JP , Kastritis PL , Bonvin AM , Vangone A . PRODIGY: a web server for predicting the binding affinity of protein–protein complexes. Bioinformatics. 2016;32(23):3676‐3678. 27503228 10.1093/bioinformatics/btw514

[33] Vangone A , Bonvin AM . Contacts‐based prediction of binding affinity in protein–protein complexes. Elife. 2015;4:e07454. 26193119 10.7554/eLife.07454 PMC4523921

[34] Kastritis PL , Rodrigues JPGLM , Folkers GE , Boelens R , Bonvin AMJJ . Proteins feel more than they see: fine‐tuning of binding affinity by properties of the non‐interacting surface. J Mol Biol. 2014;426(14):2632‐2652. 24768922 10.1016/j.jmb.2014.04.017

[35] Tina KG , Bhadra R , Srinivasan N . PIC: protein interactions calculator. Nucleic Acids Res. 2007;35:W473‐W476. (Web Server issue). 17584791 10.1093/nar/gkm423 PMC1933215

[36] Goedeke L , Fernández‐Hernando C . Regulation of cholesterol homeostasis. Cell Mol Life Sci. 2012;69(6):915‐930. 22009455 10.1007/s00018-011-0857-5 PMC11114919

[37] Ansari M , Ahmad N , Haqqi TJB . oxidative stress and inflammation in osteoarthritis pathogenesis: role of polyphenols. Biomed Pharmacother. 2020;129:110452. 32768946 10.1016/j.biopha.2020.110452 PMC8404686

[38] Kumar S , Adjei I , Brown S , Liseth O , Sharma BJB . Manganese dioxide nanoparticles protect cartilage from inflammation‐induced oxidative stress. Biomaterials. 2019;224:119467. 31557589 10.1016/j.biomaterials.2019.119467 PMC7025913

[39] Vassalli G , Büeler H , Dudler J , von Segesser LK , Kappenberger L . (AAV) vectors achieve prolonged transgene expression in mouse myocardium and arteries in vivo: a comparative study with adenovirus vectors. Int J Cardiol. 2003;90(2‐3):229‐238. 12957756 10.1016/s0167-5273(02)00554-5

[40] Palomeque J , Chemaly ER , Colosi P , et al. Efficiency of eight different AAV serotypes in transducing rat myocardium in vivo. Gene Ther. 2007;14(13):989‐997. 17251988 10.1038/sj.gt.3302895

[41] Kristensen LS , Andersen MS , Stagsted LVW , et al. The biogenesis, biology and characterization of circular RNAs. Nat Rev Genet. 2019;20(11):675‐691. 31395983 10.1038/s41576-019-0158-7

[42] Chen X , Han P , Zhou T , et al. circRNADb: a comprehensive database for human circular RNAs with protein‐coding annotations. Sci Rep. 2016;6:34985. 27725737 10.1038/srep34985 PMC5057092

[43] Pan C , Ding H , Dong YJC . Extracellular matrix protein patterns guide human chondrocytes adhesion and alignment characterized by vimentin and matrilin‐3. Biointerfaces. 2013;102:730‐736. 23107951 10.1016/j.colsurfb.2012.09.005

[44] Juarez M , Bang H , Hammar F , et al. Identification of novel antiacetylated vimentin antibodies in patients with early inflammatory arthritis. Ann Rheum Dis. 2016;75(6):1099‐1107. 26160441 10.1136/annrheumdis-2014-206785 PMC4893102

[45] Ospelt C , Bang H , Feist E , et al. Carbamylation of vimentin is inducible by smoking and represents an independent autoantigen in rheumatoid arthritis. Ann Rheum Dis. 2017;76(7):1176‐1183. 28183721 10.1136/annrheumdis-2016-210059 PMC5530349

[46] Armaos A , Colantoni A , Proietti G , Rupert J , Tartaglia GG . catRAPID omics v2.0: going deeper and wider in the prediction of protein–RNA interactions. Nucleic Acids Res. 2021;49(W1):W72‐W79. 34086933 10.1093/nar/gkab393 PMC8262727

[47] Pang K , Park J , Ahn SG , et al. RNF208, an estrogen‐inducible E3 ligase, targets soluble Vimentin to suppress metastasis in triple‐negative breast cancers. Nat Commun. 2019;10(1):5805. 31862882 10.1038/s41467-019-13852-5 PMC6925134

[48] Li Y , Xie P , Lu L , et al. An integrated bioinformatics platform for investigating the human E3 ubiquitin ligase–substrate interaction network. Nat Commun. 2017;8(1):347. 28839186 10.1038/s41467-017-00299-9 PMC5570908

[49] Huang PL . A comprehensive definition for metabolic syndrome. Dis Model Mech. 2009;2(5‐6):231‐237. 19407331 10.1242/dmm.001180 PMC2675814

[50] Moon S‐H , Huang C‐H , Houlihan SL , et al. p53 represses the mevalonate pathway to mediate tumor suppression. Cell. 2019;176(3):564‐580. 10.1016/j.cell.2018.11.011 PMC6483089 30580964

[51] Chu B‐B , Liao Y‐C , Qi W , et al. Cholesterol transport through lysosome–peroxisome membrane contacts. Cell. 2015;161(2):291‐306. 25860611 10.1016/j.cell.2015.02.019

[52] Shibuya Y , Chang CC , Chang T‐Y . ACAT1/SOAT1 as a therapeutic target for Alzheimer's disease. Future Med Chem. 2015;7(18):2451‐2467. 26669800 10.4155/fmc.15.161 PMC4976859

[53] Li H , George DM , Jaarsma RL , Mao X . Metabolic syndrome and components exacerbate osteoarthritis symptoms of pain, depression and reduced knee function. Ann Transl Med. 2016;4(7):133. 27162783 10.21037/atm.2016.03.48 PMC4842398

[54] Haj‐Mirzaian A , Mohajer B , Guermazi A , et al. Statin use and knee osteoarthritis outcome measures according to the presence of heberden nodes: results from the osteoarthritis initiative. Radiology. 2019;293(2):396‐404. 31502936 10.1148/radiol.2019190557

[55] Valdes AM , Zhang W , Muir K , et al. Use of statins is associated with a lower prevalence of generalised osteoarthritis. Ann Rheum Dis. 2014;73(5):943‐945. 24347568 10.1136/annrheumdis-2013-204382 PMC3995213

[56] Schwager JL , Nevitt MC , Torner J , et al. Association of serum low‐density lipoprotein, high‐density lipoprotein, and total cholesterol with development of knee osteoarthritis. Arthritis Care Res. 2022;74(2):274‐280. 10.1002/acr.24455 PMC8054264 32961029

[57] Zhang K , Ji Y , Dai H , et al. High‐density lipoprotein cholesterol and apolipoprotein A1 in synovial fluid: potential predictors of disease severity of primary knee osteoarthritis. Cartilage. 2021;13:1465S‐1473S. 33870758 10.1177/19476035211007919 PMC8808802

[58] Farnaghi S , Prasadam I , Cai G , et al. Protective effects of mitochondria‐targeted antioxidants and statins on cholesterol‐induced osteoarthritis. FASEB J. 2017;31(1):356‐367. 27737897 10.1096/fj.201600600R

[59] Gong Z , Wang K , Chen J , et al. CircZSWIM6 mediates dysregulation of ECM and energy homeostasis in ageing chondrocytes through RPS14 post‐translational modification. Clin Transl Med. 2023;13(1):e1158. 36604982 10.1002/ctm2.1158 PMC9816529

[60] Haudenschild DR , Chen J , Pang N , et al. Vimentin contributes to changes in chondrocyte stiffness in osteoarthritis. J Orthop Res. 2011;29(1):20‐25. 20602472 10.1002/jor.21198 PMC2976780

[61] Eiring AM , Harb JG , Neviani P , et al. miR‐328 functions as an RNA decoy to modulate hnRNP E2 regulation of mRNA translation in leukemic blasts. Cell. 2010;140(5):652‐665. 20211135 10.1016/j.cell.2010.01.007 PMC2924756

[62] Fabbri M , Paone A , Calore F , et al. MicroRNAs bind to Toll‐like receptors to induce prometastatic inflammatory response. Proc Natl Acad Sci USA. 2012;109(31):E2110‐E2116. 22753494 10.1073/pnas.1209414109 PMC3412003

