# Supplementary material for: Circular RNA circARPC1B functions as a stabilisation enhancer of Vimentin to prevent high cholesterol‐induced articular cartilage degeneration
Source: Clin Transl Med. 2023 Sep 22;13(9):e1415. doi: 10.1002/ctm2.1415 (PMC10517209; doi:10.1002/ctm2.1415)
Supplement: Supplementary file 1 — Supporting Information [file CTM2-13-e1415-s001.docx]

**Additional file 1**

**Circular RNA circARPC1B functions as a stabilization enhancer of Vimentin to prevent cholesterol-induced articular cartilage degeneration**

Jiarui Li^1*^, Xiang Li^1*^, Shengji Zhou^1*^, Yuxin Wang^1^, Tiantian Ying^1^, Quan Wang^1^, Yizheng Wu^2#^, Fengchao Zhao^1#^

**Supplementary materials and methods**

*Kellgren-Lawrence grade*

Grade 0 (normal knee): The knee is completely normal on x-ray. There is no narrowing of the joint space, no osteophytes (bone spurs) osteosclerosis or other bone changes.

Grade I: There is suspected narrowing of the joint space in the knee (narrowing of the joint space implies cartilage wear). There is a possibility of osteochondrosis (a bony bump on the edge of the knee joint), but it is mild.

Grade II: Definite presence of a small bony residue on the knee radiograph and suspicious narrowing of the joint space.

Grade III: Radiographs with moderate bony encumbrances, well-defined joint space narrowing, and subchondral bone sclerosis

Grade IV: radiographs with a large number of bony encumbrances, severe joint space narrowing, and significant subchondral bone sclerosis.

*Measurement of total cholesterol content*

1. Sample processing

The cartilage tissue from the tibial plateau of mice was homogenized in an ice bath by adding extract solution (isopropyl alcohol，(0.1 g cartilage/ 500 uL isopropyl alcohol)). 10,000 g, centrifuged at 4°C for 10 min, and the supernatant was placed on ice for measurement; Add 1mL of extract solution to 5 million cells, and ultrasonic break the cells in ice bath (power 300w, ultrasonic 2 seconds, interval 3 seconds, total time 3min); then centrifuge at 10,000g for 10min at 4℃, and put the supernatant on ice to be measured. Then centrifuge the cells at 10,000g for 10min at 4℃, take the supernatant and put it on ice to be measured; Plasma and joint fluids can be measured directly.

1. Measurement steps

The 50 μmol/mL cholesterol standard solution was diluted with the extract to obtain 2.5, 2, 1.25, 0.625, 0.3125, 0.15625 μmol/mL standard solution was set aside. The assay solution (180ul) and the sample (20ul) were added into the 96-well plate, mixed thoroughly, and incubated at 37℃ for 15min. The absorbance at 500 nm was measured by a microplate reader (Bio-Tek Instruments, Winooski, VT, USA).

1. Calculation of total cholesterol content

Drawing of standard curve: A standard curve was established based on the concentration (x, μmol/mL) and absorbance ΔA standard (y, ΔA standard) of the standard tube. Based on the standard curve, the ΔA determination (y, ΔA determination) was brought into the formula to calculate the sample concentration (x, μmol/mL).

Calculation of total cholesterol: Liquid volume calculation: TC content (μmol/dL) = x × 100; Calculated by sample mass: TC content (μmol/g mass) = x × V extraction ÷ W = x ÷ W; Calculated by cell number: TC content (μmol/10^4^ cell) = x x V extraction ÷ 500 = 0.002x

*Divergent primers for circRNA PCR*

Sequences of circRNAs were retrieved from the RNA-seq anlysis and compared in circbase, circbank and circAtlas. The sequence at the 5' end of the circRNA is transferred to the tail at the 3' end, and the junction between the 3' end and the 5' end is at the junction site of the circRNA. Use the web tool primer blast in NCBI ([Primer designing tool (nih.gov)](https://www.ncbi.nlm.nih.gov/tools/primer-blast/index.cgi?LINK_LOC=BlastHome)) for primers design and make sure the primers are designed on both sides of the junction site. The absolute value of the difference between "Tm" in "Sense" and "Tm" in "Anti-Sense" is not greater than 2.5, and "GC%" = 40%~70%. Primers specificity was verified by qPCR assay, and Sanger sequencing determined that the PCR amplified fragments contained junction sites.

*Generation of AAV expressing circARPC1B*

The full-length circARPC1B DNA sequence was cloned into the adeno-associated virus serotype 2 (AAV2) vector pHBAAV-CMV-circ-EF1-GFP (Hanbio Co. Ltd, Shanghai, China). The AAV2 vector pHBAAV-CMV-circ-EF1-GFP was used as a negative control. CircARPC1B or control plasmids were co-transfected with packaging plasmids (pAAV-RC and pHelper) into HEK-293T cells using Lipofectamine 3000 (Invitrogen, Carlsbad, CA, USA). After 72 h, the culture media were collected, and AAV was purified by Adenovirus purification kit (V1469-01, Biogima, San Diego, USA). The viral titre was determined using RT-qPCR.

*Cell Counting Kit-8 assay*

1 X 10^5^ chondrocytes were added to each well of a 96-well plate and cultivated for 48 h with cholesterol (Selleck). The CCK8 reagent (10 uL) was then added to each well and cultured for an hour. The absorbance at 450 nm was measured by a microplate reader (Bio-Tek Instruments).

*RNA isolation and library construction for RNA-seq*

Total RNA was isolated from the sample using TRIzol (Invitrogen), according to the manufacturer's instructions. The extracted RNA was treated with DNase to remove any genomic DNA contamination, followed by purification steps to obtain high-quality RNA suitable for downstream applications. Construction of chain specific articles using the method of removing ribosomal RNA (circRNA library building and increasing the process of removing linear RNA). Firstly, ribosomal RNA was removed from the total RNA, followed by linear RNA removal using RNase R enzyme. The remaining circRNA was broken into short fragments of 250-300 bp, using fragmented RNA as a template and random oligonucleotides as primers to synthesize the first strand of cDNA. Subsequently, the second strand of cDNA was synthesized using dNTPs (dUTP, dATP, dGTP, and dCTP) as raw materials. The purified double stranded cDNA undergoes end repair, A-tailed addition, and sequencing connections. AMPure XP beads were used to screen cDNA of around 350-400 bp. Use the USER enzyme to degrade the second strand of cDNA containing U, and finally perform PCR amplification to obtain the library. Next, perform preliminary quantification using Qubit and dilute the library to 1ng/ul; Then, Agilent 2100 bioanalyzer was used to detect the insert size of the library. The insert size distribution was approximately 250-300 bp, which was expected. After the Insert size meets expectations, the qPCR method is used to accurately quantify the effective concentration of the library. The effective concentration of the library is>2nM, ensuring the quality of the sample library. Samples were sequenced by NovaSeq 6000 (Illumina) after quality inspection, and paired-end reads of 150nt length were obtained. Sequence reads were trimmed for adaptor seauence/ow-gualty secuence using cutadapt(v2.7). Trimmed sequence reads were aligned to human genome (ensembl GRCh38) with bwa (v0.7.17). CIRI (V2.0.6) was applied to identify circRNAs and estimate the junction reads as circRNA counts and junction reads counts normalization were performed using DESeq2 .

*RNA fluorescent in situ hybridization (FISH)*

The cells were seeded in 6 well plates with sterile glass covers and allowed to culture overnight. Cells were then fixed with 4% paraformaldehyde (PFA) for 20 min at 25 °C and dehydrated with 70% ethanol at 4 °C for 1 h. Slides were hybridized for 14–16 h at 37 °C. Hybridization buffer for RNA FISH dissolved the probes at a concentration of 20 nM. Following overnight hybridization, the slides were washed with 10% formamide/2×SSC at 37 °C on an oscillator for 30 min, and subsequently washed with PBS and 0.1% (v/v) Tween 20 three times. The cover glass was restained with DAPI, fixed with ProLong Gold anti-fading reagents, reagents, and observed using a fluorescence microscope. For in vivo FISH, tissue sections were deparaffinized, rehydrated, and permeabilized by 0.8% pepsin treatment at 37°C for 30 min before hybridization.

*RNA pull-down assay (RPD) and* *Mass Spectrometry (MS)*

For RNA pull-down assay, the biotinylated probe solution (4 μg) was denatured at 90°C for 2 min and incubated with pre-cooled RNA structure buffer to form RNA secondary structures. Next, streptavidin magnetic beads (Invitrogen) were incubated with the mixture at 25°C for 30 min. 10^7^ C28/I2 cells were lysed at 4℃ and centrifuged with 15000g for 15min, supernatant fractions collected and mixed with the probe-bead mixture. RNase inhibitor (5 μL), EDTA (5 μL) and EGTA (2.5 μL) were added, followed by incubation for 2 h and elution at 37°C for 2 h.

LC-MS/MS analysis was performed on a Q Exactive mass spectrometer (Thermo Scientific) that was coupled to Easy nLC (Proxeon Biosystems, now Thermo Fisher Scientific) for 30/60/120/240 min (determined by project proposal). The mass spectrometer was operated in positive ion mode. MS data was acquired using a data-dependent top20 method dynamically choosing the most abundant precursor ions from the survey scan (300–1800 m/z) for HCD fragmentation. Automatic gain control (AGC) target was set to 1e6, maximum inject time to 50 ms，and number of scan ranges to 1. Dynamic exclusion duration was 30.0 s. Survey scans were acquired at a resolution of 70,000 at m/z 100 and resolution for HCD spectra was set to 17,500 at m/z 100, Automatic gain control (AGC) target was set to 1e5,isolation width was 1.5 m/z, microscans to 1, and maximum inject time to 50 ms. Normalized collision energy was 27 eV and the underfill ratio, which specifies the minimum percentage of the target value likely to be reached at maximum fill time, was defined as 0.1%. The instrument was run with peptide recognition mode enabled. MS/MS spectra were searched using MASCOT engine (Matrix Scientific, SC, USA) against a nonredundant International Protein Index arabidopsis sequence database v3.85 (released at September 2011; 39679 sequences) from the European Bioinformatics Institute (http://www.ebi.ac.uk/). For protein identification, the following options were used. Peptide mass tolerance=20 ppm, MS/MS tolerance=0.1 Da, Enzyme=Trypsin, Missed cleavage=2, Fixed modification: Carbamidomethyl (C), Variable modification：Oxidation(M).

*Silver staining*

The proteins obtained from the RNA pulldown experiment (20 µg per well) were loaded onto SDS-polyacrylamide gels (10%) for electrophoresis. After being fixed with stationary liquid (50 mL ethanol, 10 mL acetic acid, and 40 mL double distilled H2O) for 1 h, the gels were washed with 30% ethanol for 10 min and washed twice with double distilled H2O. The gels were incubated with silver staining buffer for 10 min and washed twice with double-distilled H2O. Next, the gels were stained with a chromogenic agent until clear bands were visible, after which they were washed with the corresponding elimination agent.

*Co-immunoprecipitation (Co-ip)*

Cells were lysed in cold Pierce IP lysis buffer, sonicated in an ultrasonic processor, and subsequently centrifuged at 12,000 rpm 4°C for 5 min to collect the supernatant (input). An antibody specific for the target protein was added to the lysis buffer and incubated overnight at 4°C. On the second day, Pierce protein A/G magnetic beads were washed three times with

PBS and incubated with cell lysis buffer containing the indicated antibody for 1.5 hours at room temperature. The magnetic beads were washed three times with PBS containing 1% Triton X-100 and then boiled at 95°C in 1× loading buffer for 5 min. The indicated proteins were detected by Western blotting using specific antibodies.

*RNA Immunoprecipitation (RIP)*

2 × 10^7^ cells were collected and fully lysed in polysome lysis buffer containing protease inhibitors and RNA enzyme inhibitors (lysis buffer: protease inhibitors: RNA enzyme inhibitors = 1700:17:7.5). then, the DNA removal process was carried out. Cell lysate was divided into three groups (input, IP, and IgG groups). 5 μg Anti-AGO2/Vimentin/FLAG antibodies and 5 μg IgG antibody were incubated with cell lysates of IP and IgG groups, respectively, at 4 °C for 16 h. Next, the RNA-protein complexes were isolated by incubating cell lysates with the protein A/G magnetic beads at 4 °C for 1 h. After proteinase K digestion, protein-bound RNAs were extracted by phenol/chloroform/isoamyl alcohol (125:24:1). The protein-bound RNAs

were detected by qRT-PCR and assessed by %Input (%Input =2^−[ΔCtIP-(ΔCtinput-log2 Input Dilution Factor)]^, Input Dilution Factor = (Volume _Input_/ Volume _IP/IgG_)^−1^).

*Crosslinking-immunprecipitation (CLIP)*

Cells were expanded in six 15cm dishes and cultured to a density of 80% polymerization. Then, the cells were placed on ice and placed under 365 nm UV light for cross-linking for 10 min. Cells were harvested and lysed with 1ml lysate (protease inhibitor and a final concentration of 1 mM DTT were added prior to use) on ice for 10 min. The supernatant was centrifuged and RNase T1 stock was added to a final concentration of 1 U/μL and incubated at 22°C for 10 min. Take 50 μL of the supernatant as the Input group and store it at -80°C. Take 500 ul as the IP group and 500 ul as the IgG group. 30ul Protein A/G magnetic beads were added with 10ug Vimentin or IgG; incubated at 4°C for 2h in inversion and then left on a magnetic rack for 1min to remove the supernatant. The antibody-bead mixture was resuspended with 500ul of cell lysate; and incubated at 4°C for 3 h. After 5 washes with 1mL of washing solution, the magnetic beads were suspended with 100 ul of lysate and 20U of RNase-free DNase I was added; and the beads were incubated at 37°C for 15 min. Add 900ul of lysate, blow and mix and place on a magnetic stand for 3min, then remove the supernatant; then resuspend the magnetic beads with 80ul of lysate. The Input group was supplemented with 8 μL of Proteinase K to 80ul of lysate, and the IP group was supplemented with 8 μL of Proteinase K. The lysate was digested for 15 min at 55℃; Digest at 55℃ for 15 min. Add 1 mL of Trizol to the digested solution, shake vigorously for 15 s, add 200 μL of chloroform, shake vigorously again for 15 s, and place on ice for 2 min. Centrifuge at 12,000 g for 15 min at 4°C, transfer the supernatant to a new 2 mL enzyme-free tube, add an equal volume of isopropanol, and precipitate overnight at -80°C. Centrifuge at 12,000 g for 10 min at 4°C and discard the supernatant. Add 1 mL of 75% ethanol (prepared in enzyme-free water) and wash the precipitate once. centrifuge the supernatant at 8000 g for 5 min at 4°C, discard the supernatant, and air-dry it at room temperature for 10 min to evaporate all the ethanol; add 20 μL of ddH2O (RNase DNase free) to solubilize the RNA. The RNAs were detected by qRT-PCR and assessed by %Input (%Input= =2^−[ΔCtIP-(ΔCtinput-log2 Input Dilution Factor)^], Input Dilution Factor = (Volume _Input_/ Volume _IP/IgG_)^−1^).

*Plasmids and small interfering RNA (siRNA)*

For overexpression assay, chondrocytes were harvested into 6-well plates, and medium was changed once every two days. When the cell density reached 70%-90%, they were transfected with circARPC1B or VIM vector using lipofectamine 3000 (Invitrogen, Carlsbad, CA, USA) according to the manufacturer’s protocol. After two days, transfected chondrocytes were used for next step of experiment.

For shRNA transfection assay, HEK293T were harvested into 10 cm petri dishes. When the cell density reached 30%, they were transfected with 2 ug lentiviral plasmid, 1.5 ug pSPAX2 plasmid and 0.5 pMD2G plasmid using lipofectamine 3000 according to the manufacturer’s protocol. After 18-24 hours, harvested chondrocytes into 10 cm petri dishes at about 30-50% confluency and changed medium in the HEK293T plate into 5 ml fresh medium. After about 24 hours after medium change, take one plate of transfected HEK293P, 3 ml medium was taking out using 5 ml syringe. Medium was filtered through 0.45 um filter (Millipore SLHV033RB) and supplemented with 3 ml fresh medium and 10 ug/ml final concentration polybrene (gene pharma). Used this culture medium to culture chondrocytes for 24 hours and then replaced it with normal cell culture medium. Then the cells were selected with puromycin (GIBCO), and the surviving cells were continuously cultured as stable cells for next step of experiment.

For siRNA transfection assay, chondrocytes were harvested into 6-well plates, and medium was changed once every two days. When the cell density reached 70%-90%, they were transfected with siRNA using lipofectamine 3000 according to the manufacturer’s protocol. After two days, transfected chondrocytes were used for next step of experiment.

*Filipin staining for cholesterol assay*

Cartilage samples were fixed with 4% PFA and then snap frozen with liquid nitrogen. The 5-10 μm sections were incubated with fixative solution for 30 minutes at 21°C, and then incubated with hydrolysate solution for one hour at 21°C. Finally, after 10 minutes of incubation at 21 °C in the anti-interference solution, they were incubated with Filipin Staining solution 21 °C for 30 minutes in the dark. The sections were observed using a fluorescence microscope immediately after staining was completed.

*Histological Analyses*

Cartilage specimens were fixed in 4% PFA for paraffin embedding. Each paraffin-embedded cartilage sample was sectioned at 5 μm, and every tenth section was stained with 0.1% safranin O solution and 0. 1% Fast Green solution. Cartilage destruction was scored by two observers blinded to group-identifying information using the OARSI grading system [2]. OARSI score is equal to the grade times the stage score. Grade score divide into 6 levels. Therefore, grade 1.0 would have intact chondrocytes, whereas in grade 1.5, cell death by apoptosis or necrosis would be seen. Similarly, in grade 2.0, the surface discontinuity would consist of fibrillation only, whereas in grade 2.5, additionally, abrasion of the surface with loss of a portion of the superficial cartilage zone would be seen. In grade 3, penetration into the mid zone is seen by the presence of simple fissures or clefts. In grade 3.5, extension of fissures to become branched or complex fissures is noted. In grade 4, where the key feature is erosion, grade 4.0 represents superficial zone delamination (loss of the superficial zone only), whereas grade 4.5 shows excavation into the mid zone. In grade 5, where denudation is a key feature, grade 5.0 represents presence of a bone surface which consists of intact calcified cartilage or sclerotic bone, whereas in grade 5.5 at the denuded surface, reparative fibrocartilaginous tissue or new bone formation is observed. In grade 6, grade 6.0, deformation of the joint geometry at the joint margins is seen, whereas in grade 6.5, the extent of remodeling is such that both the joint margins and force bearing areas show deformation changes.
