# Supplementary material for: Circular RNA circARPC1B functions as a stabilisation enhancer of Vimentin to prevent high cholesterol‐induced articular cartilage degeneration
Source: Clin Transl Med. 2023 Sep 22;13(9):e1415. doi: 10.1002/ctm2.1415 (PMC10517209; doi:10.1002/ctm2.1415)
Supplement: Supplementary file 3 — Supporting Information [file CTM2-13-e1415-s004.docx]

**Additional file 3**

**Circular RNA circARPC1B functions as a stabilization enhancer of Vimentin to prevent cholesterol-induced articular cartilage degeneration**

Jiarui Li^1*^, Xiang Li^1*^, Shengji Zhou^1*^, Yuxin Wang^1^, Tiantian Ying^1^, Quan Wang^1^, Yizheng Wu^2#^, Fengchao Zhao^1#^

**Supplemental Figure:**

Supplemental Figure 1. Supplementary materials for Fig. 1

Supplemental Figure 2. Supplementary materials for Fig. 2

Supplemental Figure 3. Supplementary materials for Fig. 3

Supplemental Figure 4. Supplementary materials for Fig. 4

Supplemental Figure 5. Supplementary materials for Fig. 5

Supplemental Figure 6. Supplementary materials for Fig. 6.7

**Figure S1**


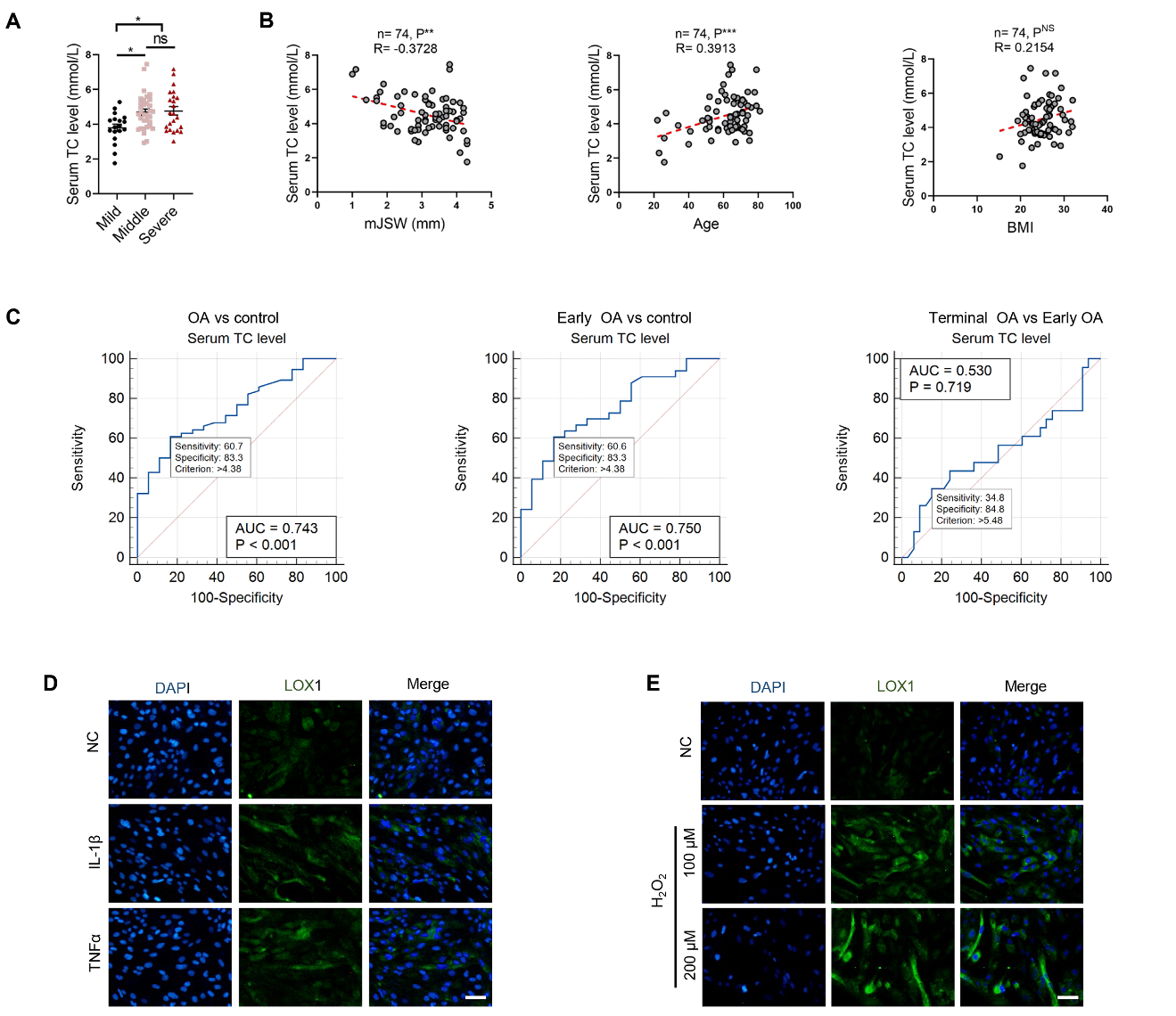


**Fig. S1 Supplementary materials for Fig. 1**

**A.** The level of serum total cholesterol of patients with mild, middle, and severe OA. **B.** Pearson correlation analysis between serum total cholesterol level and mJSW of patients with OA, age, and BMI. **C.** Receiver operating characteristic (ROC) curve for total cholesterol in synovial fluids discriminating control and OA, control and early OA, early OA and terminal OA. Control, early OA, and terminal OA represent patients with mild OA, middle OA, and severe OA, respectively, according to the KL grades. AUC, area under the ROC curve. **D-E.** Representative immunofluorescence images (n=3) of LOX1 in C28/I2 cells treated with hydrogen peroxide, IL-1β or TNFα for 36h. Scale bar, 100 μm.

**Figure S2**


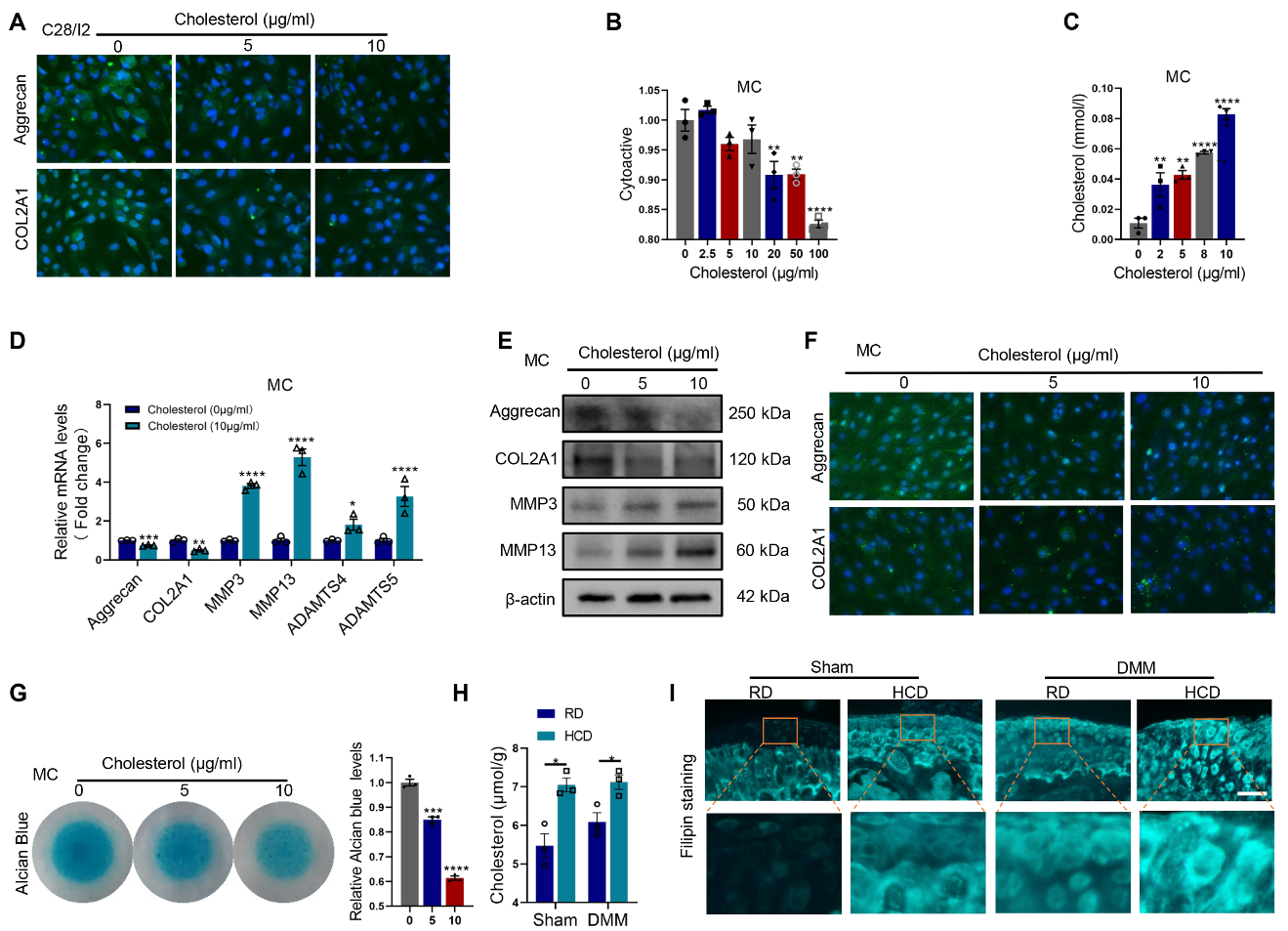


**Fig. S2 Supplementary materials for Fig. 2**

**A.** Representative immunofluorescence images (n=3) of Aggrecan and COL2A1 in C28/I2 cells treated with hydrogen peroxide, IL-1β or TNFα for 48 h. Scale bar, 100 μm. **B.** Cholesterol impact on MCs viability was detected by CCK-8 assay at 48 h (n=3). **C.** Total cholesterol level in MCs treated with 0, 2, 5, 8, 10 ug/ml cholesterol for 48 h (n=3). **D.** The mRNA expression level of COL2A1, Aggrecan, MMP3, MMP13, ADAMTS4, and ADAMTS5 in MCs treated with cholesterol for 48 h (n=3). **E.** COL2A1, Aggrecan, MMP3, MMP13, ADAMTS4, and ADAMTS5 protein levels in MCs treated with 0, 2.5, 5, 10 ug/ml cholesterol for 48 h (n=3). **F.** Representative immunofluorescence images (n=3) of Aggrecan and COL2A1 in MCs treated with hydrogen peroxide, IL-1β or TNFα for 48 h. Scale bar, 100 μm. **G.** Micromass culture and quantification of MCs treated with 0, 5, 10 ug/ml cholesterol for 7 days (n=3). **H.** The cartilage TC of HCD mice compared with RD mice in Sham and DMM group (n = 3). **I.** Representative images of Filipin staining of cartilage in four study group. Scale bar, 50 μm. The results were presented as mean ± SEM. *P＜0.05, **P＜0.01, *** P＜0.005 and **** P＜0.001 as compared with the control group.

**Figure S3**

**
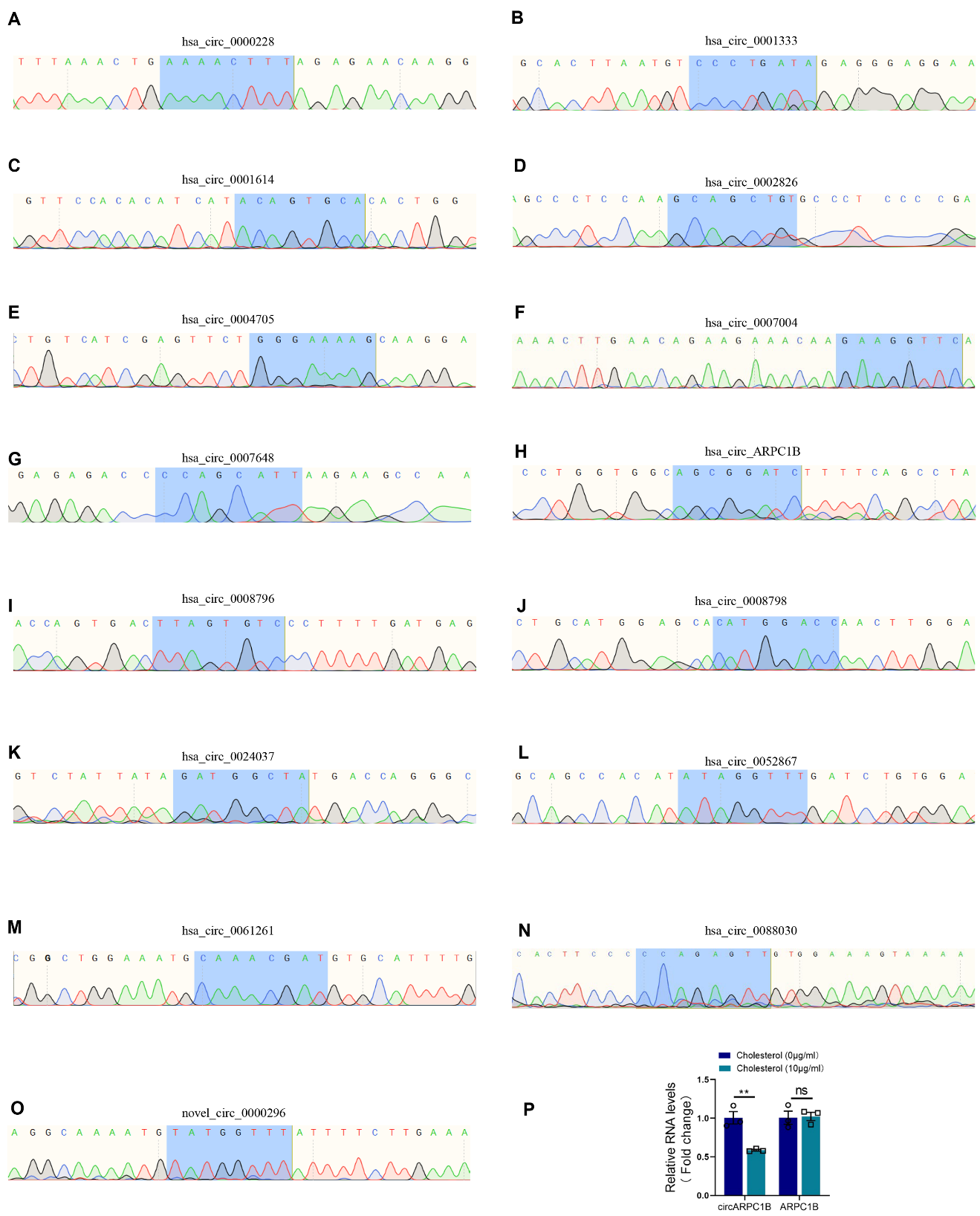
**

**Fig. S3 Supplementary materials for Fig. 3**

1. Sanger Sequencing of hsa_circ_0000228. **B.** Sanger Sequencing of hsa_circ_0001333. **C.** Sanger Sequencing of hsa_circ_0001614. **D.** Sanger Sequencing of hsa_circ_0002826. **E.** Sanger Sequencing of hsa_circ_0004705. **F.** Sanger Sequencing of hsa_circ_0007004. **G.** Sanger Sequencing of hsa_circ_0007648. **H.** Sanger Sequencing of hsa_circ_ARPC1B. **I.** Sanger Sequencing of hsa_circ_0008796. **J. S**anger Sequencing of hsa_circ_0008798. **K. S**anger Sequencing of hsa_circ_0024037. **L. S**anger Sequencing of hsa_circ_0052867. **M. S**anger Sequencing of hsa_circ_0061261. **N. S**anger Sequencing of hsa_circ_0088030. **O. S**anger Sequencing of novel_circ_0000296. **P.** circARPC1B and mARPC1B expression in C28/I2 cells (n=3). The blue area shows the backspliced junction site. *P < 0.05, **P＜0.01, *** P＜0.005 and **** P＜0.001 as compared with the control group.

**Figure S4**

**
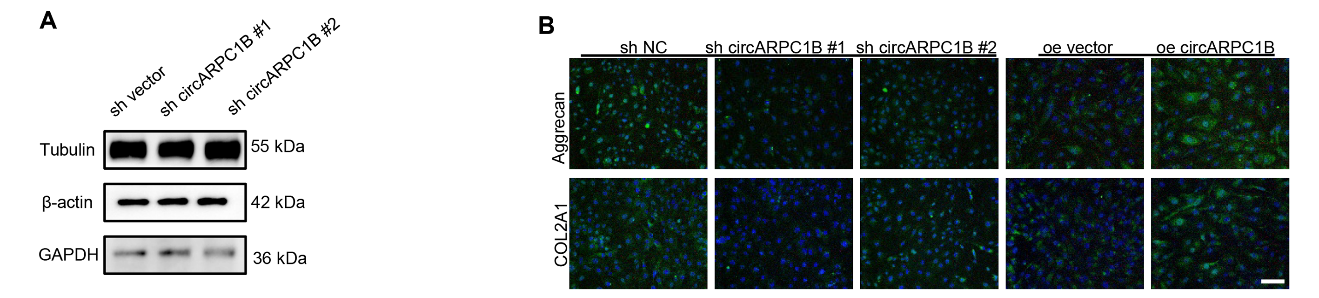
 Supplementary materials for Fig. 4**

**A.**  β-actin，Tubulin and GAPDH protein levels in C28/I2 cells after circARPC1B knockdown (n=3). **B.** Representative immunofluorescence images (n=3) of Aggrecan and COL2A1 in C28/I2 cells after circARPC1B knockdown and overexpression, scale bar, 100 μm.

**Figure S5**


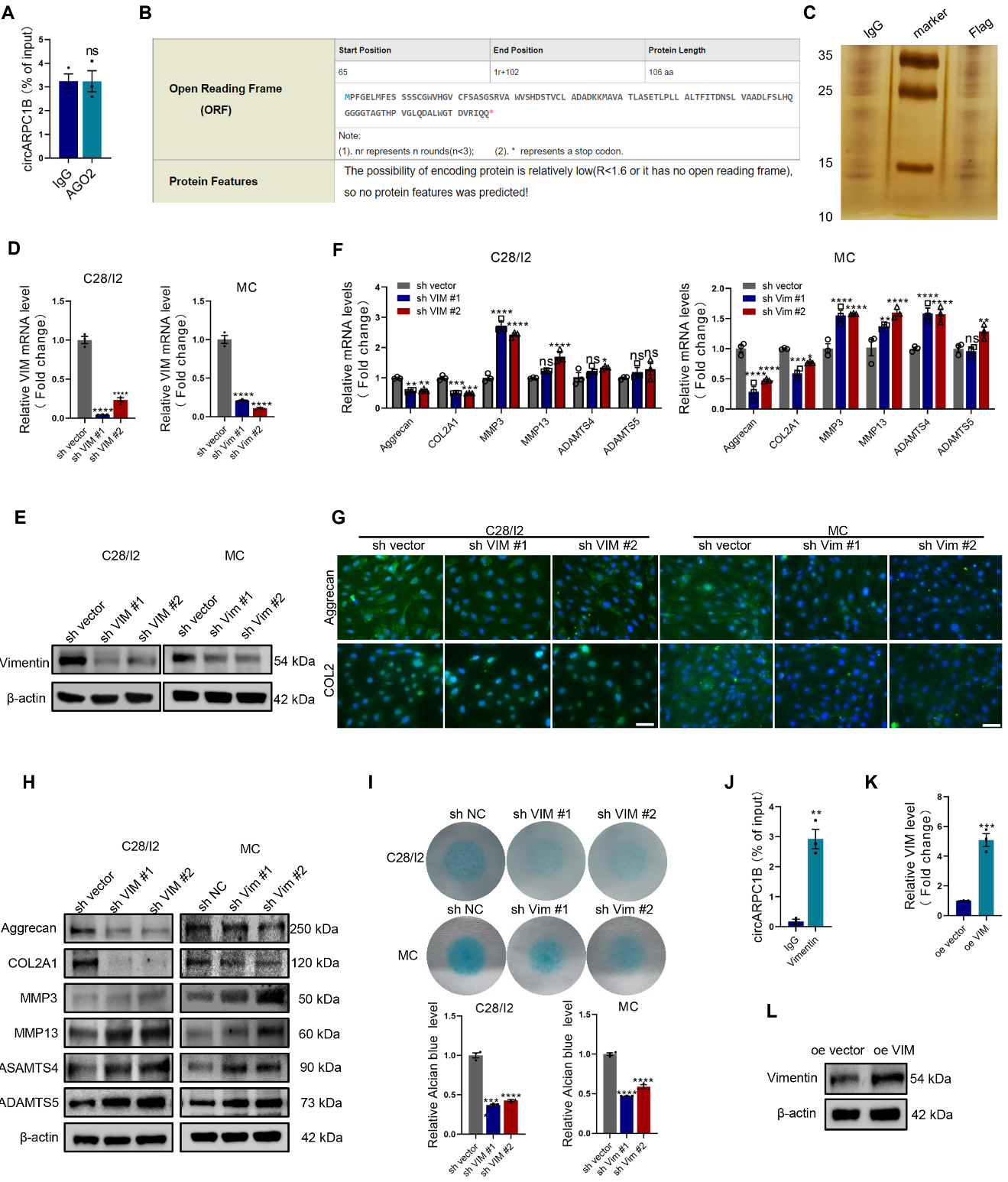


**Fig. S5 Supplementary materials for Fig. 5**

**A.** circARPC1B expression in AGO2 RIP assay. **B.** Bioinformatic analysis of circARPC1B by circRNADb software. **C.** Silver staining of proteins interacting with igG or Flag in C28/I2 cells transfected with circARPC1B-Flag vector. **D.** VIM mRNA expression in C28/I2 cells and MCs transfected with VIM shRNAs (n=3). **E.** Vimentin protein expression in C28/I2 cells and MCs transfected with VIM shRNAs (n=3). **F.** The mRNA expression level of COL2A1, Aggrecan, MMP3, MMP13, ADAMTS4, and ADAMTS5 in C28/I2 cells after vimentin knockdown (n=3). **G.** Representative immunofluorescence images (n=3) of Aggrecan and COL2A1 in C28/I2 cells and MCs after vimentin knockdown, scale bar, 100 μm. **H.** COL2A1, Aggrecan, MMP3, MMP13, ADAMTS4, and ADAMTS5 protein levels in C28/I2 cells and MCs after Vimentin knockdown (n=3). **I.** Micromass culture and quantification of C28/I2 cells and MCs after Vimentin knockdown for 7 days (n=3). **J.** Vimentin-circARPC1B interaction detected by CLIP assay (n=3). **K.** VIM mRNA expression in C28/I2 cells transfected with Vimentin overexpression plasmid (n=3). **L.** Vimentin protein expression in C28/I2 cells transfected with Vimentin overexpression plasmid (n=3). The results were represented as mean ± SEM. *P < 0.05, **P＜0.01, *** P＜0.005 and **** P＜0.001 as compared with the control group.

**Figure S6**


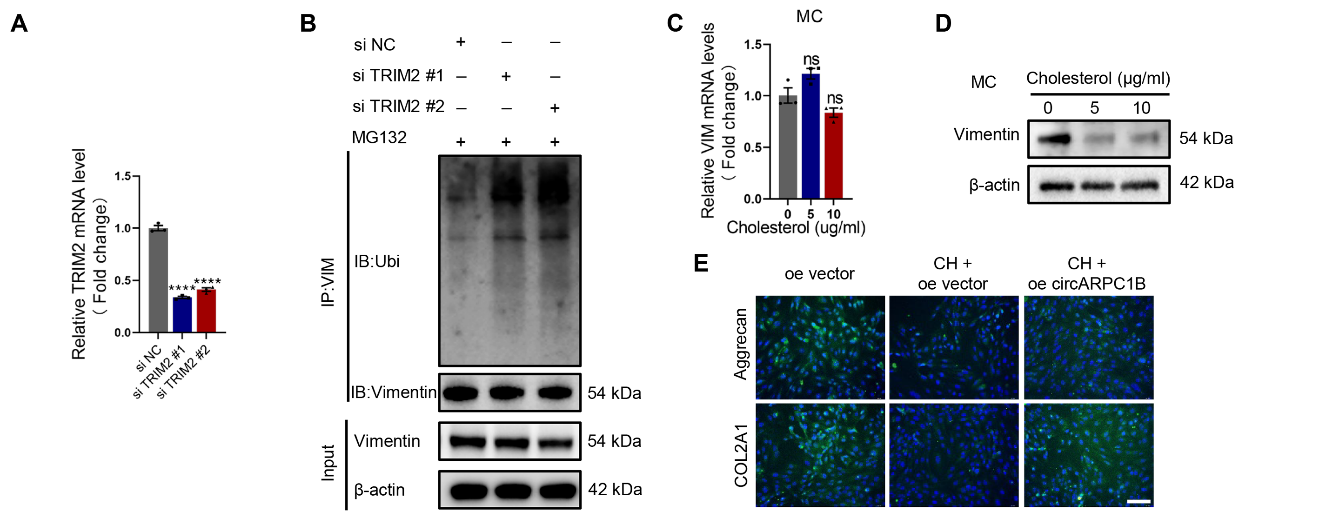


**Fig. S6 Supplementary materials for Fig. 6.7**

**A.** TRIM2 mRNA expression in C28/I2 cells transfected with TRIM2 siRNA (n=3). **B.** Effect of TRIM2 knockdown on vimentin ubiquitylation (n=3). **C.** VIM mRNA expression in MCs treated with 0, 5, 10 ug/ml cholesterol (n=3). **D.** Vimentin protein expression in MCs treated with 0, 5, 10 ug/ml cholesterol (n=3). **E.** Representative immunofluorescence images (n=3) of the effect of circARPC1B overexpression on Aggrecan and COL2A1 in C28/I2 cells treated with 10 ug/ml cholesterol, scale bar, 100 μm. The results were represented as mean ± SEM. *P < 0.05, **P＜0.01, *** P＜0.005 and **** P＜0.001 as compared with the control group.
