# Supplementary material for: Circular RNA circARPC1B functions as a stabilisation enhancer of Vimentin to prevent high cholesterol‐induced articular cartilage degeneration
Source: Clin Transl Med. 2023 Sep 22;13(9):e1415. doi: 10.1002/ctm2.1415 (PMC10517209; doi:10.1002/ctm2.1415)
Supplement: Supplementary file 4 — Supporting Information [file CTM2-13-e1415-s002.docx]

**Additional file 4**

**Circular RNA circARPC1B functions as a stabilization enhancer of Vimentin to prevent cholesterol-induced articular cartilage degeneration**

Jiarui Li^1*^, Xiang Li^1*^, Shengji Zhou^1*^, Yuxin Wang^1^, Tiantian Ying^1^, Quan Wang^1^, Yizheng Wu^2#^, Fengchao Zhao^1#^

**Supplemental Table:**

Supplemental Table 1. The information of patients.

Supplemental Table 2. Primers and sequences used in this study.

Supplemental Table 3. Top 20 CircARPC1B-binding proteins identified by mass spectrometry (ranked by prot_score).

Supplemental Table 4. Top 30 VIM predicted E3 ligases by UbiBrowser (ranked by score).

**Table S1.** **The information of patients.**

| 7 clinical cartilage tissue at KL stage 0 | | | | | | | | |
| --- | --- | --- | --- | --- | --- | --- | --- | --- |
| Gender | Age (year) | Height (m) | Weight (kg) | BMI | mJSW (mm) | Serum Total cholesterol (mmol/L) | Total cholesterol in joint fluid (mmol/L) | History of statin use (Yes or No) |
| Female | 26 | 1.58 | 50.5 | 20.23 | 3.8 | 3.17 | 0.067 | No |
| Male | 40 | 1.63 | 60 | 22.58 | 4.3 | 2.81 | 0.066 | No |
| Female | 26 | 1.64 | 55 | 20.45 | 4.3 | 1.76 | 0.078 | No |
| Male | 23 | 1.74 | 78 | 25.76 | 4.1 | 2.3 | 0.073 | No |
| Male | 50 | 1.65 | 62 | 22.77 | 3.7 | 3.7 | 0.069 | No |
| Male | 41 | 1.72 | 75 | 25.35 | 4.2 | 3.57 | 0.078 | No |
| Male | 27 | 1.70 | 62 | 21.45 | 4.2 | 4.64 | 0.073 | No |
|  | | | | | | | | |
| 11 clinical cartilage tissue at KL stage 1 | | | | | | | | |
| Gender | Age (year) | Height (cm) | Weight (kg) | BMI | mJSW (mm) | Serum total cholesterol (mmol/L) | Total cholesterol in joint fluid (mmol/L) | History of statin use (Yes or No) |
| Male | 51 | 1.75 | 65 | 21.22 | 4.2 | 4.91 | 0.068 | No |
| Female | 67 | 1.60 | 65 | 25.39 | 3.6 | 4.38 | 0.066 | No |
| Female | 57 | 1.50 | 52.5 | 23.33 | 3.7 | 3.64 | 0.072 | No |
| Male | 52 | 1.70 | 72.5 | 25.09 | 4.1 | 4.29 | 0.077 | No |
| Female | 22 | 1.61 | 55 | 21.22 | 3.4 | 4.22 | 0.079 | No |
| Male | 34 | 1.80 | 90 | 27.78 | 3.7 | 3.91 | 0.069 | No |
| Male | 52 | 1.75 | 70 | 22.86 | 3.3 | 3.83 | 0.064 | No |
| Male | 48 | 1.69 | 70 | 24.51 | 4.0 | 4.2 | 0.070 | No |
| Male | 52 | 1.65 | 73 | 26.81 | 4.0 | 4.14 | 0.095 | No |
| Female | 64 | 1.52 | 60 | 25.97 | 3.7 | 3.61 | 0.074 | No |
| Female | 72 | 1.60 | 60 | 23.44 | 3.9 | 5.28 | 0.077 | No |
|  | | | | | | | | |
| 12 clinical cartilage tissue at KL stage 2 | | | | | | | | |
| Gender | Age (year) | Height (cm) | Weight (kg) | BMI | mJSW (mm) | Serum total cholesterol (mmol/L) | Total cholesterol in joint fluid (mmol/L) | History of statin use (Yes or No) |
| Female | 70 | 1.60 | 60 | 23.44 | 3.9 | 3.73 | 0.072 | No |
| Female | 64 | 1.57 | 65.5 | 26.57 | 3.7 | 4.44 | 0.076 | No |
| Male | 74 | 1.65 | 74 | 27.21 | 3.1 | 4.5 | 0.078 | No |
| Female | 53 | 1.60 | 71 | 27.73 | 4.3 | 3.01 | 0.082 | No |
| Male | 68 | 1.60 | 54 | 21.09 | 3.5 | 4.58 | 0.073 | No |
| Female | 62 | 1.49 | 64 | 28.83 | 3.7 | 3.74 | 0.069 | No |
| Female | 51 | 1.59 | 60 | 23.73 | 3.4 | 4.35 | 0.078 | No |
| Male | 65 | 1.65 | 65 | 23.88 | 3.8 | 7.17 | 0.081 | No |
| Male | 66 | 1.63 | 68 | 25.59 | 3.2 | 5.19 | 0.078 | No |
| Female | 76 | 1.44 | 55 | 26.52 | 3.7 | 4.96 | 0.081 | No |
| Male | 70 | 1.67 | 64.5 | 23.13 | 4.1 | 5.22 | 0.081 | No |
| Female | 64 | 1.64 | 60 | 22.31 | 3.8 | 7.46 | 0.077 | Yes |
|  | | | | | | | | |
| 21 clinical cartilage tissue at KL stage 3 | | | | | | | | |
| Gender | Age (year) | Height (cm) | Weight (kg) | BMI | mJSW (mm) | Serum total cholesterol (mmol/L) | Total cholesterol in joint fluid (mmol/L) |  |
| Female | 68 | 1.55 | 55 | 22.90 | 3.1 | 4.21 | 0.081 | No |
| Female | 68 | 1.60 | 54 | 21.09 | 3.5 | 4.23 | 0.082 | No |
| Male | 67 | 1.67 | 78 | 27.97 | 2.9 | 2.93 | 0.078 | No |
| Female | 67 | 1.56 | 55 | 22.60 | 3.4 | 4.56 | 0.081 | No |
| Female | 71 | 1.56 | 65 | 26.71 | 3.9 | 4.69 | 0.079 | No |
| Male | 75 | 1.70 | 65 | 22.49 | 3.1 | 3.47 | 0.079 | No |
| Female | 61 | 1.55 | 69 | 28.72 | 2.8 | 5.71 | 0.082 | No |
| Male | 68 | 1.69 | 80 | 28.01 | 2.9 | 3.88 | 0.083 | No |
| Female | 67 | 1.65 | 65 | 23.88 | 3.2 | 3.75 | 0.082 | No |
| Male | 57 | 1.68 | 73 | 25.86 | 3.7 | 3.70 | 0.078 | No |
| Male | 71 | 1.60 | 68 | 26.56 | 3.6 | 5.42 | 0.083 | No |
| Female | 56 | 1.62 | 78 | 29.72 | 3.8 | 4.73 | 0.083 | No |
| Male | 62 | 1.64 | 75 | 27.88 | 3.1 | 5.39 | 0.084 | No |
| Female | 59 | 1.62 | 70 | 26.67 | 3.3 | 5.92 | 0.081 | Yes |
| Male | 64 | 1.70 | 70 | 24.22 | 3.1 | 4.13 | 0.082 | No |
| Female | 74 | 1.58 | 73 | 29.24 | 3.3 | 5.07 | 0.082 | No |
| Female | 67 | 1.58 | 66 | 26.44 | 3.5 | 3.84 | 0.072 | No |
| Female | 81 | 1.53 | 48 | 20.50 | 3.7 | 4.75 | 0.088 | No |
| Male | 70 | 1.65 | 70.8 | 26.01 | 3.2 | 5.12 | 0.098 | No |
| Female | 52 | 1.50 | 66 | 29.33 | 3.1 | 5.59 | 0.083 | Yes |
| Male | 67 | 1.68 | 70 | 24.80 | 3.7 | 5.48 | 0.087 | No |
|  | | | | | | | | |
| 23 clinical cartilage tissue at KL stage 4 | | | | | | | | |
| Gender | Age (year) | Height (cm) | Weight (kg) | BMI | mJSW (mm) | Serum total cholesterol (mmol/L) | Total cholesterol in joint fluid (mmol/L) |  |
| Male | 72 | 1.65 | 60 | 22.04 | 1.9 | 3.85 | 0.090 | No |
| Male | 64 | 1.64 | 59 | 21.94 | 2.1 | 3.86 | 0.099 | No |
| Female | 63 | 1.60 | 65 | 25.39 | 2.3 | 3.54 | 0.094 | No |
| Female | 72 | 1.66 | 68 | 24.68 | 2.7 | 3.56 | 0.086 | No |
| Male | 71 | 1.60 | 68 | 26.56 | 3.0 | 4.66 | 0.091 | No |
| Female | 71 | 1.52 | 60 | 25.97 | 2.4 | 4.62 | 0.092 | No |
| Female | 79 | 1.58 | 70 | 28.04 | 1.1 | 7.17 | 0.080 | Yes |
| Male | 73 | 1.60 | 60 | 23.44 | 2.9 | 4.2 | 0.089 | No |
| Female | 68 | 1.50 | 55 | 24.44 | 1.7 | 5.52 | 0.081 | No |
| Male | 78 | 1.60 | 55 | 21.48 | 1.8 | 5.85 | 0.099 | No |
| Female | 71 | 1.45 | 52 | 24.73 | 2.7 | 3.68 | 0.080 | No |
| Male | 65 | 1.80 | 80 | 24.69 | 2.9 | 3.64 | 0.083 | No |
| Male | 75 | 1.77 | 75 | 23.94 | 2.9 | 3.02 | 0.088 | No |
| Male | 66 | 1.64 | 67 | 24.91 | 2.3 | 5.59 | 0.085 | No |
| Female | 61 | 1.66 | 88 | 31.93 | 1.9 | 4.04 | 0.077 | No |
| Male | 64 | 1.70 | 56 | 19.38 | 2.2 | 4.39 | 0.080 | No |
| Male | 79 | 1.73 | 80 | 26.73 | 2.4 | 5.06 | 0.074 | No |
| Male | 57 | 1.67 | 65 | 23.31 | 1.7 | 5.33 | 0.086 | No |
| Female | 60 | 1.65 | 80 | 29.38 | 1.9 | 6.31 | 0.085 | No |
| Female | 57 | 1.55 | 48 | 19.98 | 2.8 | 3.61 | 0.082 | No |
| Female | 63 | 1.63 | 55 | 20.70 | 1.0 | 6.89 | 0.073 | Yes |
| Male | 74 | 1.64 | 73 | 27.14 | 2.5 | 5.87 | 0.086 | No |
| Female | 1 | 1.64 | 65 | 24.17 | 1.4 | 5.39 | 0.098 | No |

**Table S2. Primers and sequences used in this study.**

| Primers for qPCR | | | |
| --- | --- | --- | --- |
| Human β-actin | F | AGAGCTACGAGCTGCCTGAC | |
|  | R | AGCACTGTGTTGGCGTACAG | |
| Human MMP3 | F | CCTACAAGGAGGCAGGCAAG | |
|  | R | CCCGTCACCTCCAATCCAAG | |
| Human MMP13 | F | TCGGCCACTCCTTAGGTCTT | |
|  | R | AAGTGGCTTTTGCCGGTGTA | |
| Human ADAMTS4 | F | GTCCCATGTGCAACGTCAAG | |
|  | R | ATGCGGCCATCTTGTCATCT | |
| Human ADAMTS5 | F | GGGCACTGGCTACTATGTGG | |
|  | R | CGTCACAGCCAGTTCTCACA | |
| Human COL2A1 | F | ATGACAATCTGGCTCCCAAC | |
|  | R | GAACCTGCTATTGCCCTC | |
| Human Aggrecan | F | GGGACCTGCAAGGAGACAGAG | |
|  | R | TCAATCTCACACAGGTCCCCTTC | |
| Mouse β-actin | F | AGCCATGTACGTAGCCATCC | |
|  | R | CTCTCAGCTGTGGTGGTGAA | |
| Mouse Aggrecan | F | CACTGTCAAAGCACCATGCC | |
|  | R | TAGGCTGGCTCCCATTCAGT | |
| Mouse COL2A1 | F | CACGCATGAGCCGAAGCTA | |
|  | R | GGGTTTCCACGTCTCACCA | |
| Mouse MMP3 | F | ACTGTGTCCCAAGGAGAGGAG | |
|  | R | AAACCATCTACACAGTTCAGACAC | |
| Mouse MMP13 | F | CAAGCAGTTCCAAAGGCTACA | |
|  | R | TAGGGCTGGGTCACACTTCT | |
| Mouse ADAMTS4 | F | TTGTTCTCCCAGTCACCCTCC | |
|  | R | AGCCTGGGACTAAAGATAGGCA | |
| Mouse ADAMTS5 | F | ATGCAGCCATCCTGTTCACC | |
|  | R | AAGGCCAAGTAGATGCCCAATTT | |
| Human LOX1 | F | GGCATGCAATTATCCCAGGTG | |
|  | R | TGCCAGATCCAGTCTTGCG | |
| Human HMGCR | F | TTGGTGATGGGAGCTTGTTGT | |
|  | R | CGAGCCAGGCTTTCACTTCT | |
| Human CH25H | F | GGTCATCTTCTCCATCACCACA | |
|  | R | TCCATGTCGAAGAGTAGCAGG | |
| Human CYP7B1 | F | TCTCTTTGCCGCCACCTTAC | |
|  | R | AGGCTTTCGCTGATAATCGG | |
| Human SREBP2 | F | AGCTGACCCTGGGAGACATC | |
|  | R | TGACTTGCAGAGTTGGAGCC | |
| Human SCAP | F | GCAGCACAGGCATCAAGTTC | |
|  | R | CCCAAAGTGCCTGACAGATGAT | |
| Human INSIG1 | F | CATCTTTTCCTCCGCCTGGT | |
|  | R | ATGTCCACCAAAGGCCCAAA | |
| Human INSIG2 | F | GCGGGGGATTTCTGGTAGG | |
|  | R | ACACCGCATTACACTGGACC | |
| Human LDLR | F | TGTTCCCACGTCTGCAATGA | |
|  | R | GGATGAGGCTGGTGTACTCG | |
| Human ABCG1 | F | TGTCTGATGGCCGCTTTCTC | |
|  | R | GGACCCATAATGGCCACCAA | |
| Human NPC1 | F | GACTCCGGAAGGCAAACAGA | |
|  |  | ATCGCTCTTCAGTGGCACAA | |
| Human NPC2 | F | AGTCTATCTGGGGGTGAGAGG | |
|  | R | GCTGGACCTTCCTTACTCCG | |
| Human LXRα | F | ACTGATGTTCCCACGGATGC | |
|  | R | CACAGTGTTAGCGAGGGCT | |
| Human LXRβ | F | CACAGTCACAGTCGCAGTCA | |
|  | R | TCGGAGAAGGAGCGTTTGTT | |
| Human U6 | F | CTCGCTTCGGCAGCACA | |
|  | R | AACGCTTCACGAATTTGCGT | |
| Human ARPC1B | F | CGTCGACTGCCCAGAGTCC | |
|  | R | TCGTGCACCTTGGTCCATTT | |
| Hsa_circ_0007940 (circARPC1B) | F | CTGGCCTCTGAAACACTACCA | |
|  | R | GCTGGCTGAGAAACAGACG | |
| Mouse Vimentin | F | TGAGATCGCCACCTACAGGA | |
|  | R | TTGCGCTCCTGAAAAACTGC | |
| Human Vimentin | F | GGACCAGCTAACCAACGACA | |
|  | R | AAGGTCAAGACGTGCCAGAG | |
| Hsa_circ_0000228 | F | GAGGTGTGGGGTGTGAGAAC | |
|  | R | TGTCAAGCAGACAGTAGCCAA | |
| Hsa_circ_0001333 | F | TCCTCAAGGACTACAGTATGCAC | |
|  | R | TACGTTAGGCAAACGCCCAT | |
| Hsa_circ_0001614 | F | TCTACATCCCAGAAAGTTGCTGAT | |
|  | R | ACCAGTGTGCACTGTATGATGT | |
| Hsa_circ_0002826 | F | CCTGCGATGGGGCGAGAAA | |
|  | R | GGGAGAAGCGTCCACGAAA | |
| Hsa_circ_0004705 | F | ACACCATGCGCAAGAACAAG | |
|  | R | CCTTGCTTTTCCCAGAACTCG | |
| Hsa_circ_0007004 | F | CCGCAAAGCGCCGGAA | |
|  | R | CCATGTAGTCCTGTCGCTGAT | |
| Hsa_circ_0007648 | F | GTGGAGACAGTAGCTGGAACC | |
|  | R | TGGCTTCTTAAATGCTGGGGT | |
| Hsa_circ_0007940 | F | CGACTCTGGCCTCTGAAACA | |
|  | R | GCTGAAAAGATCCGCTGCCA | |
| Hsa_circ_0008796 | F | GCACAGTGATTAAACTGGGGC | |
|  | R | GCTGTTGAATCAGAATGAGGCT | |
| Hsa_circ_0008798 | F | CCGTACATCGTGGGCTTCTA | |
|  | R | TCCAAGTTGGTCCATGTGCT | |
| Hsa_circ_0024037 | F | CCCGTAGCAGGAACTCAAAGA | |
|  | R | TGAGATCTGAGTGCTCTGGC | |
| Hsa_circ_0052867 | F | CAGCTCAGCAGCCACATATAG | |
|  | R | CTCTCCCATTCCCCGAGATTC | |
| Hsa_circ_0061261 | F | TTATGAAATCTGTACCAACCAACG | |
|  | R | AGTGGCTCCAAAGTGCTTACA | |
| Novel_circ_0000296 | F | ACCATGTGGGAGGAGGAAAAT | |
|  | R | AAGCCTGGAGCCTTTTCAAGA | |
| Hsa_circ_0088030 | F | CCACTTCCCCCAGAGTTGTG | |
|  | R | AATGGAGTGCGTTGTCCAGC | |
| Divergent- β-actin | F | CAGGGCTTACCTGTACACTGA | |
|  | R | GCGCGGCGATATCATCATCC | |
| Human  SYVN1 | F | TGCGTAACATCCACACACTG | |
|  | R | AGGCTAAACCTTCTGCCTTCA | |
| Human  TRIM2 | F | GGCCACGTCAAGCAGAAAGCTGT | |
|  | R | ACTGGGATGTACAGCCACTCCTGT | |
| CircARPC1B shRNA-1 | | TGGTGGCAGCGGATCTTTT | |
| CircARPC1B shRNA-2 | | GGCAGCGGATCTTTTCAGC | |
| Vimentin shRNA-1 | | CCGGGCAGGATGAGATTCAGAATATCTCGAGATATTCTGAATCTCATCCTGCTTTTTT | |
| Vimentin shRNA-2 | | CCGGCGCCATCAACACCGAGTTCAACTCGAG-TTGAACTCGGTGTTGATGGCG-TTTTTT | |
| Mmu Vimentin shRNA-1 | | CCGGGCTTCAAGACTCGGTGGACTTCTCGAGAAGTCCACCGAGTCTTGAAGCTTTTTT | |
| Mmu Vimentin shRNA-2 | | CCGGGTGGAATCCTTGCAGGAAGAACTCGAGTTCTTCCTGCAAGGATTCCACTTTTTT | |
| SiRNAs | | | |
| Hsa_circ_0007004 si | | F | GAAACAAGAAGGUUCAAGUTT |
|  | | R | ACUUGAACCUUCUUGUUUCTT |
| Hsa_circ_0007940 si | | F | CUGGUGGCAGCGGAUCUUUTT |
|  | | R | AAAGAUCCGCUGCCACCAGTT |
| Hsa_circ_0008796 si | | F | CAGUGACUUAGUGUCCCUUTT |
|  | | R | AAGGGACACUAAGUCACUGTT |
| Hsa_SYVN1 si-1 | | F | GCAUUGUCUCUCUUAUGUU |
|  | | R | AACAUAAGAGAGACAAUGC |
| Hsa_SYVN1 si-2 | |  | CUGUACAUGGCCUUCAUGA |
|  | |  | UCAUGAAGGCCAUGUACAG |
| Hsa_TRIM2 si-1 | | F | GCAUUAAGAGCUGCAGCAA |
|  | | R | UUGCUGCAGCUCUUAAUGC |
| Hsa_TRIM2 si-2 | | F | GGUGUAGCAGUGGAUUCAA |
|  | | R | UUGAAUCCACUGCUACACC |
| Overexpressed plasmids | | | |
| Hsa_circ_0007940 | | GATCTTTTCAGCCTACATCAAGGAGGTGGAGGAACGGCCGGCA  CCCACCCCGTGGGGCTCCAAGATGCCCTTTGGGGAACTGATGT  TCGAATCCAGCAGTAGCTGCGGCTGGGTACATGGCGTCTGTTT  CTCAGCCAGCGGGAGCCGCGTGGCCTGGGTAAGCCACGACAG  CACCGTCTGCCTGGCTGATGCCGACAAGAAGATGGCCGTCGCG  ACTCTGGCCTCTGAAACACTACCACTGCTGGCGCTGACCTTCA  TCACAGACAACAGCCTGGTGGCAGCG | |
| Hsa_VIM | | gaattcATGTCCACCAGGTCCGTGTCCTCGTCCTCCTACCGCAGGA  TGTTCGGCGGCCCGGGCACCGCGAGCCGGCCGAGCTCCAGCC  GGAGCTACGTGACTACGTCCACCCGCACCTACAGCCTGGGCA  GCGCGCTGCGCCCCAGCACCAGCCGCAGCCTCTACGCCTCGTC  CCCGGGCGGCGTGTATGCCACGCGCTCCTCTGCCGTGCGCCTG  CGGAGCAGCGTGCCCGGGGTGCGGCTCCTGCAGGACTCGGTG  GACTTCTCGCTGGCCGACGCCATCAACACCGAGTTCAAGAAC  ACCCGCACCAACGAGAAGGTGGAGCTGCAGGAGCTGAATGA  CCGCTTCGCCAACTACATCGACAAGGTGCGCTTCCTGGAGCA  GCAGAATAAGATCCTGCTGGCCGAGCTCGAGCAGCTCAAGG  GCCAAGGCAAGTCGCGCCTGGGGGACCTCTACGAGGAGGAG  ATGCGGGAGCTGCGCCGGCAGGTGGACCAGCTAACCAACGAC  AAAGCCCGCGTCGAGGTGGAGCGCGACAACCTGGCCGAGGA  CATCATGCGCCTCCGGGAGAAATTGCAGGAGGAGATGCTTCA  GAGAGAGGAAGCCGAAAACACCCTGCAATCTTTCAGACAGG  ATGTTGACAATGCGTCTCTGGCACGTCTTGACCTTGAACGCA  AAGTGGAATCTTTGCAAGAAGAGATTGCCTTTTTGAAGAAAC  TCCACGAAGAGGAAATCCAGGAGCTGCAGGCTCAGATTCAGG  AACAGCATGTCCAAATCGATGTGGATGTTTCCAAGCCTGACCT  4CACGGCTGCCCTGCGTGACGTACGTCAGCAATATGAAAGTGT  GGCTGCCAAGAACCTGCAGGAGGCAGAAGAATGGTACAAAT  CCAAGTTTGCTGACCTCTCTGAGGCTGCCAACCGGAACAATG  ACGCCCTGCGCCAGGCAAAGCAGGAGTCCACTGAGTACCGG  AGACAGGTGCAGTCCCTCACCTGTGAAGTGGATGCCCTTAAA  GGAACCAATGAGTCCCTGGAACGCCAGATGCGTGAAATGGAA  GAGAACTTTGCCGTTGAAGCTGCTAACTACCAAGACACTATTG  GCCGCCTGCAGGATGAGATTCAGAATATGAAGGAGGAAATGG  CTCGTCACCTTCGTGAATACCAAGACCTGCTCAATGTTAAGAT  GGCCCTTGACATTGAGATTGCCACCTACAGGAAGCTGCTGGAA  GGCGAGGAGAGCAGGATTTCTCTGCCTCTTCCAAACTTTTCCT  CCCTGAACCTGAGGGAAACTAATCTGGATTCACTCCCTCTGGT  TGATACCCACTCAAAAAGGACACTTCTGATTAAGACGGTTGAA  ACTAGAGATGGACAGGTTATCAACGAAACTTCTCAGCATCA  CGATGACCTTGAAGATTACAAGGATGACGACGATAAGTAAgg  atcc | |
| Probes for FISH | | | |
| Cy3-Hsa circARPC1B | | 5‘-Cy3-AAAGATCCGCTGCCACCAGGC-3’ | |
| RNA pull down sequences | | | |
| CircPDE4B pull-down probe | | GATCTTTTCAGCCTACATCAAGGAGGTGGAGGAACGGCCG  GCACCCACCCCGTGGGGCTCCAAGATGCCCTTTGGGGAAC  TGATGTTCGAATCCAGCAGTAGCTGCGGCTGGGTACATGG  CGTCTGTTTCTCAGCCAGCGGGAGCCGCGTGGCCTGGGTA  AGCCACGACAGCACCGTCTGCCTGGCTGATGCCGACAAGA  AGATGGCCGTCGCGACTCTGGCCTCTGAAACACTACCACT  GCTGGCGCTGACCTTCATCACAGACAACAGCCTGGTGGCA  GCGGATCTTTTCAGCCTACATCAAGGAGGTGGAGGAA | |
| Lac Z pull-down probes | | TGGCCGTCGTTTTACAACGTCGTGACTGGGAAAACCCTGG  CGTTACCCAACTTAATCGCCTTGCAGCACATCCCCCTTTC  GCCAGCTGGCGTAATAGCGAAGAGGCCCGCACCGATCGCC  CTTCCCAACAGTTGCGCAGCCTGAATGGCGAATGGCGCCT  GATGCGGTATTTTCTCCTTACGCATCTGTGCGGTATTTCA  CACCGCATATGGTGCACTCTCAGTACAATCTGCTCTGATG CCGCATAG | |

**Table S3.** **Top 20 CircARPC1B-binding proteins identified by mass spectrometry (ranked by prot_score).**

| Number | Accession | Description | prot_score |
| --- | --- | --- | --- |
| 1 | P08670 | Vimentin OS=Homo sapiens OX=9606 GN=VIM PE=1 SV=4 | 323 |
| 2 | Q15149 | Plectin OS=Homo sapiens OX=9606 GN=PLEC PE=1 SV=3 | 323 |
| 3 | P35579 | Myosin-9 OS=Homo sapiens OX=9606 GN=MYH9 PE=1 SV=4 | 323 |
| 4 | P01040 | Cystatin-A OS=Homo sapiens OX=9606 GN=CSTA PE=1 SV=1 | 323 |
| 5 | P68871 | Hemoglobin subunit beta OS=Homo sapiens OX=9606 GN=HBB PE=1 SV=2 | 277 |
| 6 | P49756 | RNA-binding protein 25 OS=Homo sapiens OX=9606 GN=RBM25 PE=1 SV=3 | 176 |
| 7 | P62979 | Ubiquitin-40S ribosomal protein S27a OS=Homo sapiens OX=9606 GN=RPS27A PE=1 SV=2 | 135 |
| 8 | F8VZY9 | Keratin, type I cytoskeletal 18 OS=Homo sapiens OX=9606 GN=KRT18 PE=1 SV=1 | 134 |
| 9 | P04259 | Keratin, type II cytoskeletal 6B OS=Homo sapiens OX=9606 GN=KRT6B PE=1 SV=5 | 130 |
| 10 | Q0VAS5 | Histone H4 OS=Homo sapiens OX=9606 GN=HIST1H4H PE=1 SV=1 | 96 |
| 11 | P15924 | Desmoplakin OS=Homo sapiens OX=9606 GN=DSP PE=1 SV=3 | 90 |
| 12 | B4DVQ0 | cDNA FLJ58286, highly similar to Actin, cytoplasmic 2 OS=Homo sapiens OX=9606 PE=2 SV=1 | 81 |
| 13 | A0A7I2V5N4 | Elongation factor 1-alpha OS=Homo sapiens OX=9606 GN=EEF1A1 PE=1 SV=1 | 80 |
| 14 | B5BU08 | U2 small nuclear RNA auxillary factor 1 isoform a OS=Homo sapiens OX=9606 GN=U2AF1 PE=2 SV=1 | 76 |
| 15 | B3KPS3 | Tubulin alpha chain OS=Homo sapiens OX=9606 PE=2 SV=1 | 72 |
| 16 | A0A024R5C5 | Pyruvate carboxylase OS=Homo sapiens OX=9606 GN=PC PE=4 SV=1 | 70 |
| 17 | O75533 | Splicing factor 3B subunit 1 OS=Homo sapiens OX=9606 GN=SF3B1 PE=1 SV=3 | 65 |
| 18 | B3KQH1 | cDNA FLJ90452 fis, clone NT2RP3001475, highly similar to Splicing factor 3B subunit 3 OS=Homo sapiens OX=9606 PE=2 SV=1 | 64 |
| 19 | Q9Y3B4 | Splicing factor 3B subunit 6 OS=Homo sapiens OX=9606 GN=SF3B6 PE=1 SV=1 | 61 |
| 20 | P07437 | Tubulin beta chain OS=Homo sapiens OX=9606 GN=TUBB PE=1 SV=2 | 53 |

**Table S4. Top 30 VIM predicted E3 ligases by UbiBrowser (ranked by score).**

| E3 | E3GENE | HOMO | PFAM | GO | NET | MOTIF | SCORE |
| --- | --- | --- | --- | --- | --- | --- | --- |
| Q9C040 | TRIM2 | 1 | 3.17 | 1 | 1.44 | 2.12 | 0.728 |
| Q86TM6 | SYVN1 | 1 | 1 | 1.25 | 2.2 | 3.41 | 0.702 |
| O75382 | TRIM3 | 1 | 3.17 | 1.13 | 1.77 | 1 | 0.69 |
| Q13049 | TRIM32 | 1 | 3.17 | 1.13 | 1.77 | 1 | 0.69 |
| Q86YT6 | MIB1 | 1 | 1 | 1.51 | 1.44 | 2.8 | 0.687 |
| Q9UM11 | FZR1 | 1 | 1 | 1.51 | 1.84 | 2.12 | 0.684 |
| Q9UNE7 | STUB1 | 1 | 1 | 3.98 | 1.87 | 1 | 0.681 |
| Q9HCE7 | SMURF1 | 1 | 1 | 2.88 | 1.87 | 1.06 | 0.655 |
| P22681 | CBL | 1 | 1 | 2.33 | 1.55 | 1.06 | 0.625 |
| Q8WY64 | MYLIP | 1 | 1 | 1.13 | 1 | 2.8 | 0.623 |
| Q2Q1W2 | TRIM71 | 1 | 3.17 | 1 | 1 | 1 | 0.623 |
| Q13309 | SKP2 | 1 | 1 | 1 | 1.44 | 2.12 | 0.619 |
| P53804 | TTC3 | 1 | 1 | 1 | 1.44 | 2.12 | 0.619 |
| Q86YJ5 | 9-Mar | 1 | 1 | 1 | 1 | 2.8 | 0.61 |
| Q9H1K0 | ZFYVE20 | 1 | 1 | 1.51 | 1.84 | 1 | 0.609 |
| Q06587 | RING1 | 1 | 1 | 1.51 | 1.84 | 1 | 0.609 |
| P51668 | UBE2D1 | 1 | 1 | 1.51 | 1.84 | 1 | 0.609 |
| P02511 | CRYAB | 1 | 1 | 1.51 | 1.84 | 1 | 0.609 |
| Q92466 | DDB2 | 1 | 1 | 1.51 | 1.84 | 1 | 0.609 |
| O75592 | MYCBP2 | 1 | 1 | 1.51 | 1.84 | 1 | 0.609 |
| Q9NWF9 | RNF216 | 1 | 1 | 1 | 1.29 | 2.12 | 0.608 |
| Q9H992 | 7-Mar | 1 | 1 | 1 | 1.29 | 2.12 | 0.608 |
| Q9Y2K7 | KDM2A | 1 | 1 | 1.51 | 2.3 | 1 | 0.605 |
| Q15542 | TAF5 | 1 | 1 | 1.51 | 1.77 | 1 | 0.605 |
| Q14241 | TCEB3 | 1 | 1 | 1.51 | 1.77 | 1 | 0.605 |
| Q13702 | RAPSN | 1 | 1 | 1.51 | 1.77 | 1 | 0.605 |
| P61024 | CKS1B | 1 | 1 | 1.51 | 1.77 | 1 | 0.605 |
| P46736 | BRCC3 | 1 | 1 | 1.51 | 1.77 | 1 | 0.605 |
| P35227 | PCGF2 | 1 | 1 | 1.51 | 1.77 | 1 | 0.605 |
| Q15386 | UBE3C | 1 | 1 | 1.25 | 1 | 2.12 | 0.604 |
